# The Castagnoli–Cushman Reaction

**DOI:** 10.3390/molecules28062654

**Published:** 2023-03-15

**Authors:** José Luis Ramiro, Sonia Martínez-Caballero, Ana G. Neo, Jesús Díaz, Carlos F. Marcos

**Affiliations:** 1 Laboratory of Bioorganic Chemistry & Membrane Biophysics (L.O.B.O.), Departamento de Química Orgánica e Inorgánica, Facultad de Veterinaria, Universidad de Extremadura, 10003 Cáceres, Spain; 2 Departamento de Didáctica de las Ciencias Experimentales y las Matemáticas, Facultad de Formación de Profesorado, Universidad de Extremadura, 10003 Cáceres, Spain

**Keywords:** multicomponent reactions, anhydrides, Schiff bases, lactones, Perkin condensation

## Abstract

Since the first reports of the reaction of imines and cyclic anhydrides by Castagnoli and Cushman, this procedure has been applied to the synthesis of a variety of lactams, some of them with important synthetic or biological interest. The scope of the reaction has been extended to the use of various Schiff bases and anhydrides as well as to different types of precursors for these reagents. In recent years, important advances have been made in understanding the mechanism of the reaction, which has historically been quite controversial. This has helped to develop reaction conditions that lead to pure diastereomers and even homochiral products. In addition, these mechanistic studies have also led to the development of new multicomponent versions of the Castagnoli–Cushman reaction that allow products with more diverse and complex molecular structures to be easily obtained.

## 1. Introduction

The reaction of imines with cyclic anhydrides, commonly named the Castagnoli–Cushman reaction (CCR), has been used for the last 50 years for the synthesis of a wide variety of lactams. This reaction has been partially reviewed in the past, [1,2,3,4,5] but the relevant contributions made in the field in the last few years require an up-to-date account. Here, we summarise the developments and applications of the Castagnoli–Cushman reaction from its discovery until now.

**Scheme 1:**
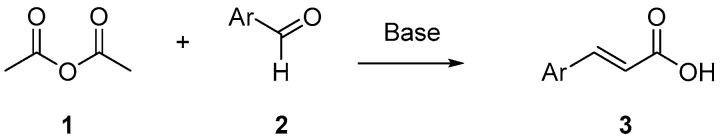
The Perkin reaction.

It has long been known that anhydrides react with different electrophiles and bifunctional reagents to yield condensation products. For example, Perkin observed 150 years ago that the condensation of anhydrides (**1**) and aldehydes (**2**) yielded α,β-unsaturated carboxylic acids (**3**; Scheme 1) [6,7]. This reaction is still used for the preparation of diverse types of compounds such as coumarins [6,8] or stilbene derivatives (**3**) [9].

**Scheme 2:**
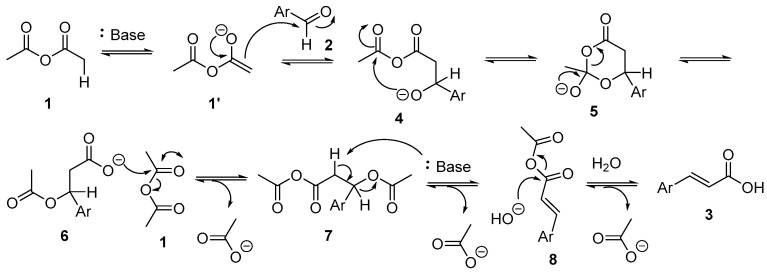
Accepted mechanism of the Perkin reaction.

The accepted mechanism for the Perkin condensation involves an initial attack of the anhydride enolate (**1′**) to the aldehyde (**2**) in a reversible pathway involving multiple intermediates (**4**–**8**; Scheme 2) [7], although some alternative mechanisms have been also proposed [10,11].

**Scheme 3:**
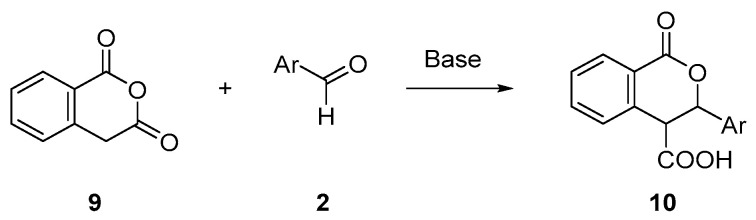
Müller synthesis of dihydroisocoumarins.

The use of enolisable cyclic anhydrides in place of lineal anhydrides was first reported by Fittig and Jayne in 1883 [12,13]. Later, Müller discovered that the homophthalic anhydride (HPA, **9**) reacts smoothly with benzaldehyde in a basic medium at room temperature to form dihydroisocoumarin (**10**; Scheme 3) [14,15,16]. This strategy has been recently implemented for the kinetic resolution of racemic α-aryl-substituted branched aldehydes in the presence of a chiral squaramide-based bifunctional catalyst [17].

**Scheme 4:**
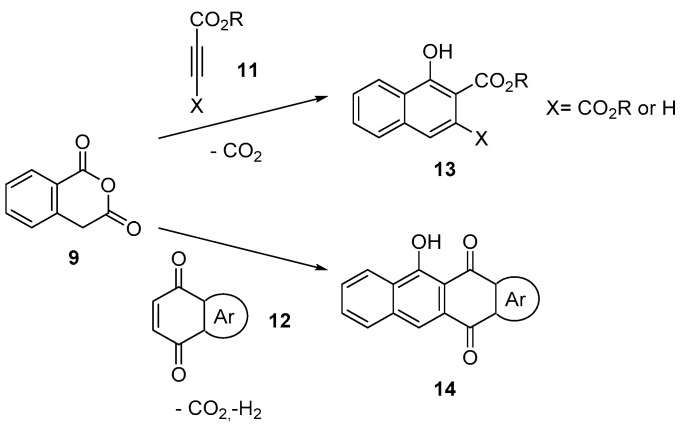
The Tamura reaction.

A related transformation of cyclic anhydrides was discovered by Tamura in 1981. He found that HPA (**9**) reacts with alkynes (**11**) and activated alkenes (**12**) at high temperatures to regioselectively yield condensed phenolic compounds (**13**) and (**14**), respectively (Scheme 4) [18]. The Tamura reaction has been efficiently used in the synthesis of bioactive compounds and natural products, especially anthracyclines [19,20]. The use of bases, such as tertiary amines, allows the reaction to take place under milder conditions and ensures that carboxylic acid intermediates do not decarboxylate [21]. On the other hand, catalytic asymmetric variants of the Tamura reaction have been also developed, resulting in excellent enantio- and diastereoselectivities [22,23,24].

**Scheme 5:**
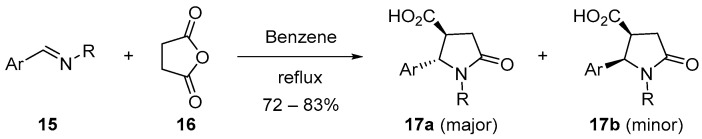
CCR with succinic anhydride.

The reaction of imines with cyclic anhydrides (CCR) was first reported in 1969 by Castagnoli, who condensed imines (**15**) with succinic anhydride (**16**) to yield 4,5-disubstituted-2-pyrrolidinones (**17**; Scheme 5) [4,25].

**Scheme 6:**
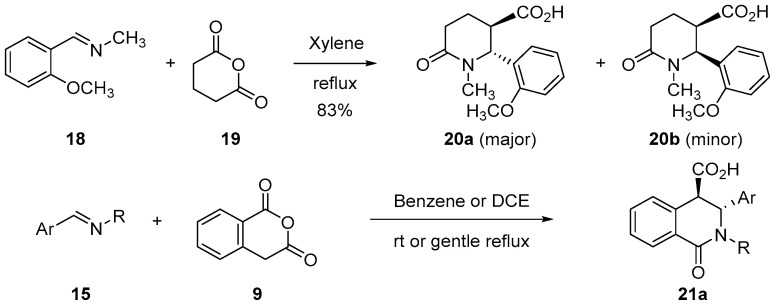
CCR with glutaric and homophthalic anhydrides.

The reaction of imines (**18**) with glutaric anhydride (**19**) to yield the diastereomeric mixture of piperidones (**20a**) and (**20b**), reported by Cushman [26], and the reaction of HPA (**9**) with imines (**15**) to afford the *trans* isoquinolones (**21a**), first reported by Haimova [27], allowed for significantly expanding the scope of the CCR and demonstrated its importance for the preparation of complex structures and natural product analogues (Scheme 6).

Classical conditions for the CCR involve the use of benzene, toluene, or xylenes at relatively high temperatures, which invariably leads to the *trans* isomer as the major product. Performing the reaction in DMF affords a higher proportion of the *cis* isomer, allowing its isolation and characterisation. However, the *trans* disubstituted lactam is still the major isomer in these conditions [28]. Other solvents have also been used, resulting in different proportions of diastereoisomers [29]. In some cases, the reaction takes place at room temperature, preferentially yielding the *cis* isomers [30,31,32,33]. Trifluoroethanol has proven to be an advantageous solvent for the CCR, allowing a variety of lactams with high diastereoselectivity for *cis*-kinetic diastereoisomers to be obtained in minutes at −40 °C [34]. In recent years, greener reaction conditions have been developed, such as solvent-free methods [35] and the use of microwaves as an alternative activation technology [36,37,38].

In summary, the CCR has found many synthetic applications. The variation of both the starting materials and the reaction conditions provides access to a large variety of products. Moreover, many post-condensation transformations have been developed that allow for a considerable increase in the molecular diversity attained through these reactions.

## 2. Mechanism

**Scheme 7:**
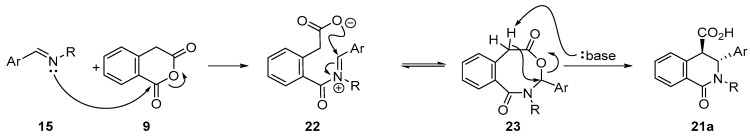
Iminolysis mechanism proposed by Castagnoli and Cushman.

The mechanism of the CCR is somewhat controversial and different alternatives have been proposed in recent years. Castagnoli and his Ph.D. student Cushman proposed that the reaction of imines (**15**) with HPA (**9**) takes place through the iminolysis of the anhydride, followed by cyclisation and rearrangement of the intermediate product (**22**; Scheme 7) [39,40]. This mechanism is consistent with the fact that the reaction has been shown to be favoured by the presence of electron-donating substituents on the Schiff base that enhance its nucleophilicity. [39,40]. Moreover, succinic anhydrides substituted with electron-withdrawing groups react very fast, which is also consistent with further enolate stabilisation on the cyclisation intermediate (**23**) [41].

**Scheme 8:**
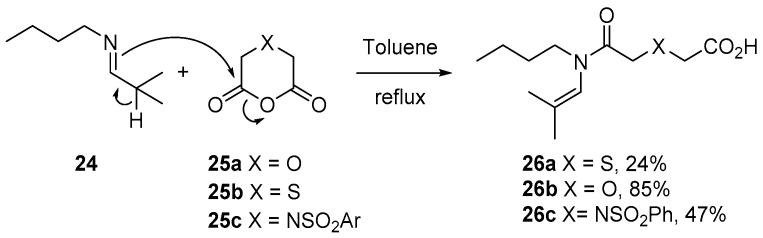
The reaction of cyclic anhydrides with enolisable imines.

This mechanism is also apparently supported by the fact that the CCR is limited to imines derived from non-enolisable aldehydes. Otherwise, enamines can be formed from the imines (**24**) and then acylated by the anhydrides (**25**), preventing the formation of the Castagnoli–Cushman adducts. In these cases, the reaction usually yields exclusively *N*-acyl enamines (**26**; Scheme 8) [42,43].

However, it has been argued that zwitterionic intermediates (**22**) shown in Scheme 7 are too high in energy, rendering the iminolysis mechanism unworkable [30,44,45].

**Scheme 9:**
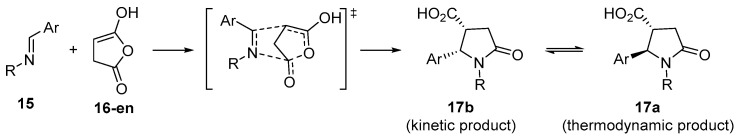
Concerted reaction mechanism proposed by Kaneti.

Based on computational studies on small model substrates, Kaneti proposed a concerted mechanism through the enol form of the cyclic anhydride (**16en**; Scheme 9) [44]. MO and DFT computation reveal the concerted mechanism would preferentially produce the *cis* diastereomer, through a less energetic TS from the imine’s *Z*-isomer, in apparent contradiction to experimental findings. Kaneti suggests that the experimental stereochemical product distribution must then result from a thermodynamically controlled process, in which the kinetic *cis* adduct (**17b**) would epimerise to yield the more stable *trans* product (**17a**), likely through an enol intermediate (Scheme 9).

Palamareva and co-workers also support a similar concerted mechanism for the CCR of imines with HPA, arguing its similarity to Tamura’s reaction, which has often been described as a [4+2] cycloaddition [46].

**Scheme 10:**
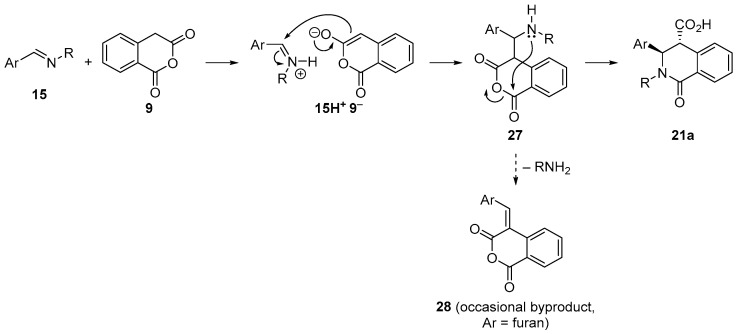
Two-step Mannich/intramolecular acylation mechanism.

More recent computational studies (considering the effect of substituents on the imine and the anhydride) and experimental findings disagree with both the concerted mechanisms and the two-step mechanisms initiated by iminolysis. In contrast, the currently most widely accepted mechanism for the CCR consists of a stepwise process involving a Mannich-type addition of the anhydride enolate to the imine, followed by the opening of the anhydride from the intramolecular attack of the intermediate amine (**27**; Scheme 10) [30,47].

**Scheme 11:**
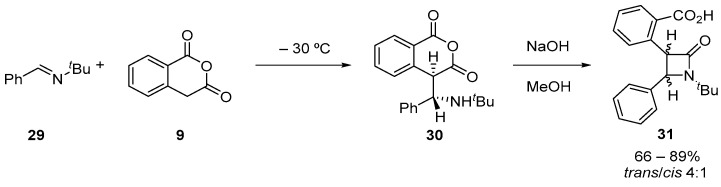
Diastereoselective synthesis of β-lactams from imines and HPA.

An experimental fact that supports this mechanism is the formation, under certain conditions, of a Knoevenagel-type alkene product (**28**), which can be explained by the elimination of an amine from intermediate **27** [48]. The occurrence of Mannich-type intermediates (**30**) was also confirmed in the reaction of HPA (**9**) with *N*-*tert*-butylbenzalimine (**29**) by its trapping in basic conditions at low temperatures to yield β-lactam products (**31**), which can be explained by the attack of the amine on the vicinal carbonyl group *(*Scheme 11) [49].

**Scheme 12:**
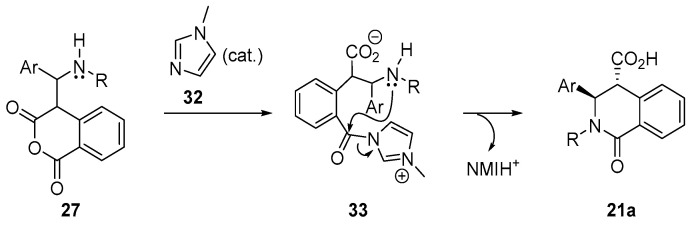
NMI catalysed the CCR.

Further experimental evidence supporting this mechanism is the fact that the CCR is catalysed by *N*-methylimidazole (**32**), which has been proposed to favour the amine acylation of the Mannich adduct intermediate (**27**) through a more reactive intermediate (**33**; Scheme 12) [45].

Shaw and Cheong proposed a Mannich reaction between the enolate of cyanosuccinic anhydride and the iminium cation which forms a pseudo-Zimmerman–Traxler transition state (TS) where the instability of zwitterionic intermediates is alleviated by hydrogen bonds. Subsequent transannular acylation would yield the final product, satisfactorily explaining both the reactivity and stereoselectivity reported by different authors for related reactions of HPAs with imines [30].

Knapp outlined different Mannich transition state possibilities for the HPA-imine reaction [49]. A transition state stabilised by the hydrogen bond between the enol and the imine nitrogen can adopt two different geometries. The aryl group can be placed either close to the benzo ring in a ‘‘chair” configuration, or away from the benzo ring in a ‘‘boat” configuration. Alternatively, an ‘‘open” transition state may also be possible, in which the favoured relative orientation of the HPA enolate and the iminium cation will depend on the relative size of the nitrogen substituent R. Knapp’s experimental results, in which the ratio of *cis* versus *trans* products increases with the increasing size of the *N*-substituents (Me < *n*-Bu < *i*-Bu < *t*-Bu), lead to the conclusion that the reaction proceeds through an open transition state that leads to a short-lived amino-anhydride intermediate (**27**).

**Figure 1:**
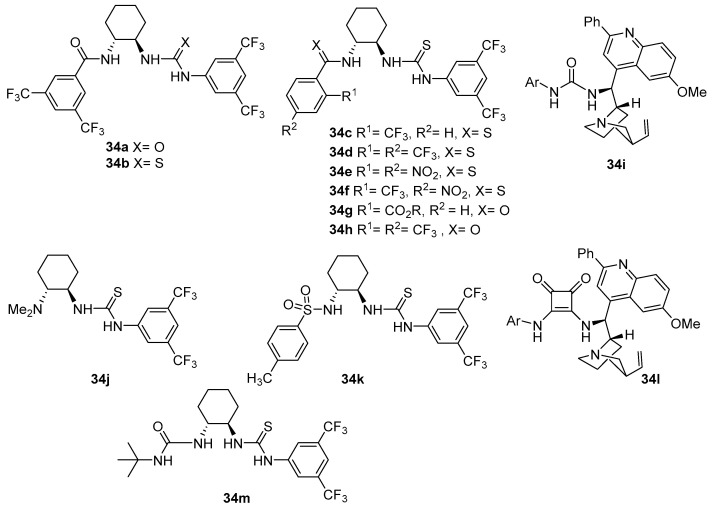
Chiral ureas and thioureas used as catalysts for the CCR.

It has been shown that CCRs can be enantiocontrolled by classical hydrogen bond donor-acceptor catalysts, such as chiral ureas and thioureas, and squaramides, as exemplified by compounds **34a-m** (Figure 1) [50,51,52].

**Scheme 13:**
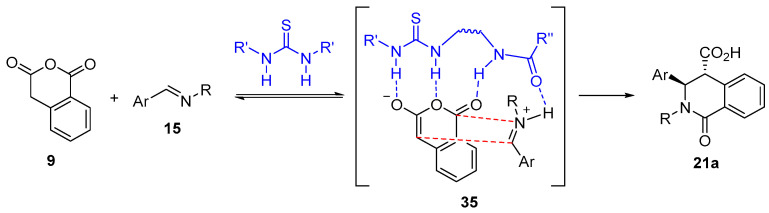
Representative mechanism of the enantioselective CCR catalysed by general urea.

DFT calculations suggest that in all cases the hydrogen bonding of the catalyst to the anhydride stabilises the formation of an ion-paired iminium-enolate intermediate. Specifically, the enolate forms interactions with the N-H of thiourea or squaramide unit of the catalyst, while the iminium cation forms additional hydrogen-bonds with a carbonyl on the urea or amide groups (Scheme 13) [50].

**Scheme 14:**
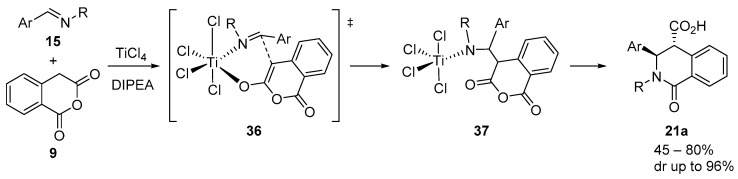
CCR catalysed by TiCl_4_.

Interestingly, Cossío et al. reported the reaction between HPA (**9**) and imines (**15**) in the presence of TiCl_4_ and diisopropyl ethyl amine to be *trans*-selective in contrast with the uncatalysed reaction made in dichloromethane at room temperature (rt), whose stereochemistry depends on the nature of the nitrogen substituent R. DFT calculations reveal that the TiCl_4_-catalysed reaction takes place through a Perkin–Mannich pathway where both the imine and the anhydride enolate are linked to the titanium in a TS (**36**) which favours the *trans* adduct (Scheme 14) [53].

## 3. Two-Component Castagnoli–Cushman Reactions (2C-CCR)

### 3.1. Anhydride Substrates

The traditional CCR involves an imine and an anhydride (commonly succinic, glutaric, or homophthalic anhydrides) allowing the synthesis of lactams with different substitution patterns. Post-condensation transformations can lead to more complex structures or a different substitution pattern. For example, β-carboxyl-γ-butyrolactams have been obtained by decarboxylative fluorination of the CCR adducts of succinic anhydrides using Selectfluor^®^ as a fluorine source [54]. However, in the last decade, several efforts have been made to increase the anhydride scope of CCR, which has allowed for the expansion of the product space covered by this reaction.

#### 3.1.1. 5-Membered Cyclic Anhydrides

**Scheme 15:**
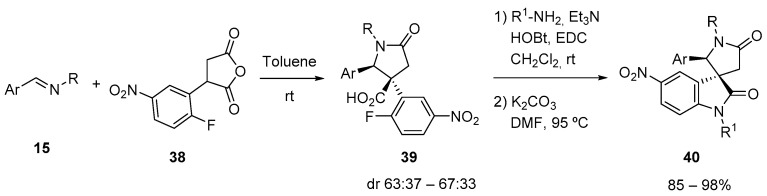
Synthesis of γ-lactams and spirobicyclic lactams from imines and succinic anhydrides (HOBt: Hydroxybenzotriazole, EDC: 1-Ethyl-3-[3-dimethylaminopropyl]carbodiimide).

An obvious extension of the CCR is the use of substituted succinic and glutaric anhydrides. Castagnoli and Cushman’s early reports already include the use of substituted anhydrides, such as 3,3-dimethylsuccinic anhydride [40] and phenylsuccinic anhydride [39]. Substitution not only introduces structural diversity but sometimes also offers synthetic advantages. Substituents that stabilise the anhydride enolate significantly facilitate the reaction. Thus, Shaw reported the smooth reaction of 2-fluoro-5-nitrophenylsuccinic anhydride (**38**) with imines (**15**) in toluene at room temperature (Scheme 15). However, as happens with other reactions at low temperatures, the diatereoselectivity is modest [29,41]. The reaction was also performed on the solid phase under microwave irradiation [29]. The resulting carboxylic acids (**39**) can be transformed into the corresponding methyl esters or amides as a mixture of diasteroisomers easily separated by column chromatography. Post-condensation transformations of Castagnoli–Cushman adducts have been employed by a number of groups to obtain complex molecules with applications in different fields. Here, biologically relevant cores, such spirooxindoles (**40**) were readily obtained from **39** in just two steps (Scheme 15) [29,41].

**Scheme 16:**
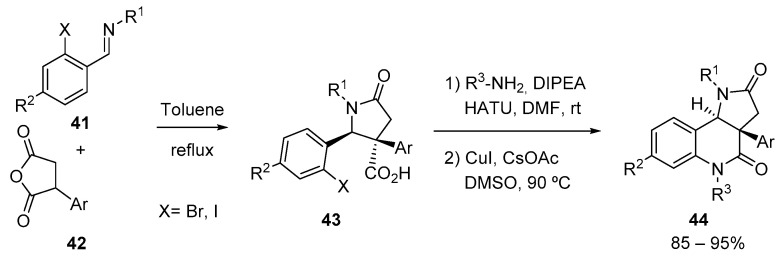
Synthesis of fused dihydroquinolones (DIPEA: *N,N*-Diisopropylethylamine, HATU: 2-(7-Aza-1*H*-benzotriazole-1-yl)-1,1,3,3-tetramethyluronium hexafluorophosphate).

Alternatively, the reaction of bromo- or iodoaryl imines (**41**) with aryl-substituted succinic anhydrides (**42**) yielded tricyclic dihydroquinolones (**44**) by an intramolecular amidation reaction of the corresponding amidolactams (**43**; Scheme 16) [29].

**Scheme 17:**
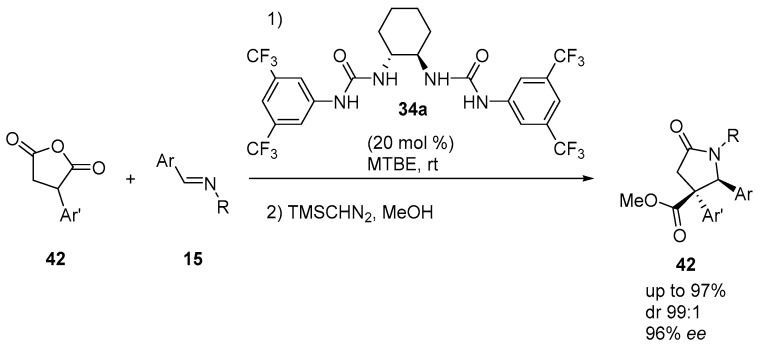
Asymmetric CCR with a phenyl-modified succinic anhydride.

Recently, aryl-substituted succinic anhydrides (**42**) have proved to be effective in CCR couplings with asymmetric bisurea catalysis (Scheme 17) [52]. The presence of a phenyl ring is essential for the correct orientation of the Mannich-type transition state, which is conducted on products with high diastereo- and enantioselelectivities and very good yields.

**Scheme 18:**
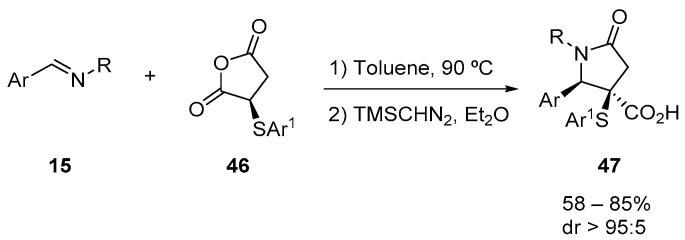
2C-CCR with arylthio-substituted succinic anhydrides.

Shaw and co-workers performed a highly diastereoselective reaction of imines (**15**) and arylthio-substituted succinic anhydrides (**46**; Scheme 18) [55]. The preferred enolisation of the thioaryl substituted α position undoubtedly determines the regio- and stereoselectivity of this reaction [42].

**Scheme 19:**
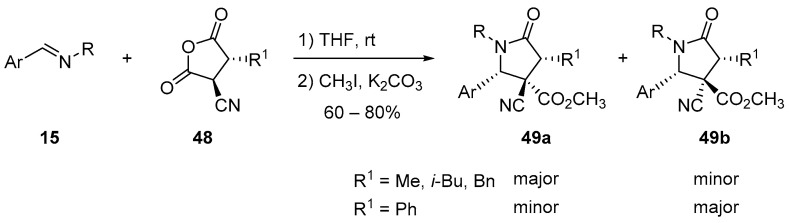
CCR with cyanosuccinic anhydrides.

Reactions with other substituted succinic anhydrides were developed by Shaw’s group [41]. Cyanosuccinic anhydrides (**48**) react smoothly at room temperature to produce highly substituted γ-lactams in high yield and with high diastereoselectivity. The resulting carboxylic acid can be further converted into its methyl ester (**49**) and reduction of the nitrile can lead to the corresponding amines by hydrogenation with Nickel-Raney catalysis. The use of enantiomerically pure alkyl-substituted cyanosuccinic anhydrides affords enantiomerically pure penta-substituted lactam products (Scheme 19) [30,31].

**Scheme 20:**
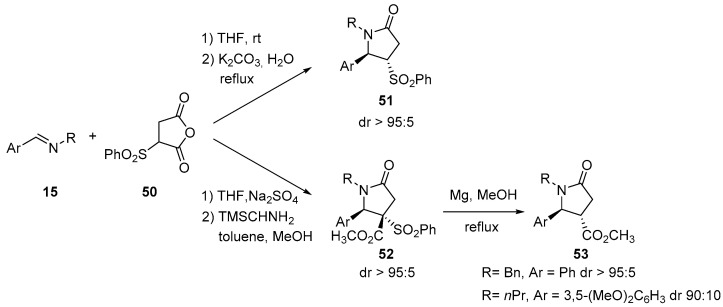
CCR with sulfone-substituted succinic anhydride.

Likewise, sulfone-substituted succinic anhydrides (**50**) produce the corresponding sulfone-substituted γ-lactam carboxylic acids that can be trapped as methyl esters (**52**) or allowed to decarboxylate under mild conditions to yield *anti*-sulfones (**51**; Scheme 20). Sulfone-substituted γ-lactam **52** can also be smoothly desulfonylated with magnesium in methanol to produce the corresponding lactam **53** in good yields [56].

**Scheme 21:**
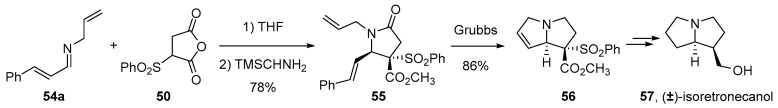
Synthesis of (±)-isoretronecanol from cinnamaldehyde and allylamine.

This strategy was employed for the preparation of the alkaloid (±)-isoretronecanol (**57**) from the imine **54a** and anhydride **50**. The ester (**55**) was then subjected to a ring-closing metathesis to produce the pyrrolizidine scaffold (**56**) that yields (±)-isoretronecanol (**57**) through two alternative routes in three or two reaction steps, respectively (Scheme 21) [56].

Surprisingly, other heteroatom-substituted succinic anhydrides, such as those containing -OCH_3_, -OPh, -NHCHO, and 1-pyrrolyl, did not react or yield unidentified products [55].

**Scheme 22:**
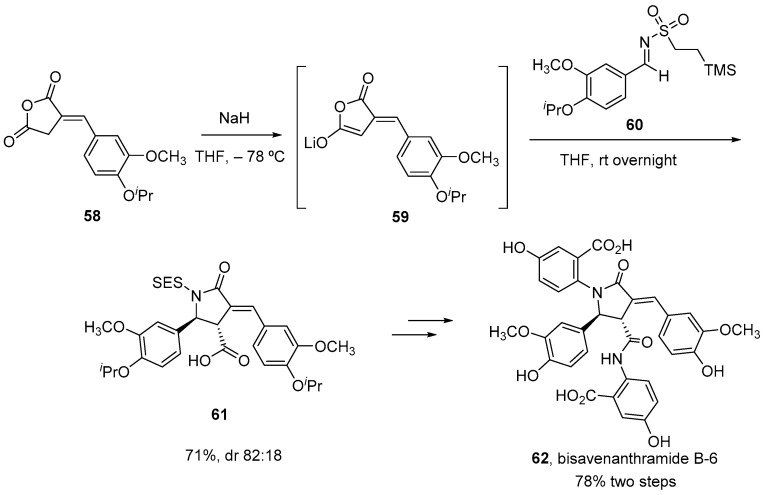
Synthesis of bisavenanthramide (B-6) by an anionic CCR (SES: 2-(trimethylsilyl)ethanesulfonyl).

An anionic version of the Castagnoli–Cushman reaction was developed by Shaw and was used for the synthesis of the phytoalexin bisavenanthramide B-6 (**62**) [57]. In this reaction, 3-benzylidenedihydrofuran-2,5-dione (**58**) is treated with sodium hydride at a low temperature to yield the corresponding enolate (**59**). This reacts with *N*-sulfonyl protected imine (**60**) to produce a *trans*-δ-lactam intermediate (**61**) which is further transformed into the desired bisavenanthramide B-6 (**62**) by a double Buchwald *N*-arylation (Scheme 22).

**Scheme 23:**
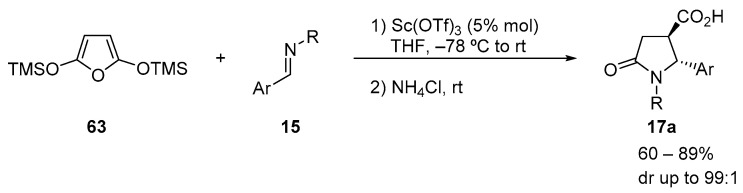
Mukaiyama-related CCR.

Likewise, a Mukaiyama-aldol type reaction has been described by Pohmakotr and Reutrakul, where 2,5-bis(trimethysilyloxy)furan (**63**) reacts with imines (**15**), employing Sc(OTf)_3_ as a catalyst, to produce the lactam **17a** (Scheme 23) [58].

**Scheme 24:**
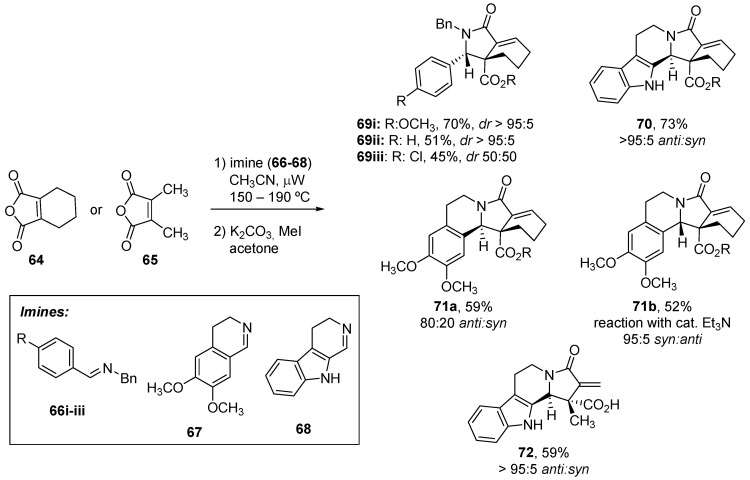
CCR with alkyl-substituted maleic anhydrides.

An interesting attempt to incorporate new anhydrides into the CCR chemical arsenal was made by Shaw and co-workers, who reacted alkyl-substituted maleic anhydrides (**64**–**65**) with the imines **66**–**68**, providing valuable information about the behaviour of maleic anhydride analogues in CCR [36]. Allylic stabilisation of the intermediate enolates facilitates the reaction of alkyl-substituted maleic anhydrides (**64**–**65**) with cyclic (**67**–**68**) or acyclic imines (**66**, R = H), providing a synthesis of unsaturated lactams **69**–**72**. The kinetic *anti*-diestereoisomer is usually formed as the major product. Interestingly, diastereoselectivity is reversed when the reaction is performed in the presence of triethylamine and triethylammonium hydrochloride, and regiochemistry seems to be defined by the alkyl region of the anhydride (Scheme 24).

**Scheme 25:**
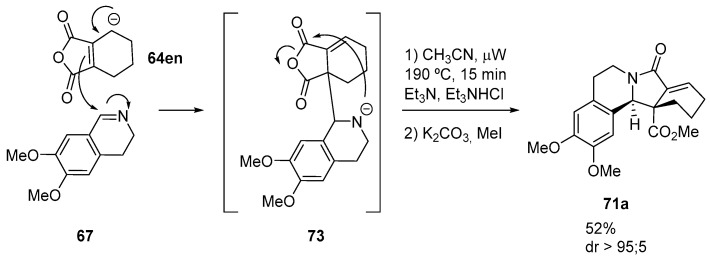
A possible mechanism for the formation of polycyclic lactam **66**.

According to the currently accepted mechanism of the CCR [49,59], polycyclic lactams **71** are formed by a first attack of the allylic enolate of anhydride **64** to the imine **67**, yielding the intermediate **73**. Then, cyclisation by the intramolecular attack of the nitrogen on the distal carbonyl of the anhydride results in the final product (**71a**; Scheme 25) [36].

**Scheme 26:**
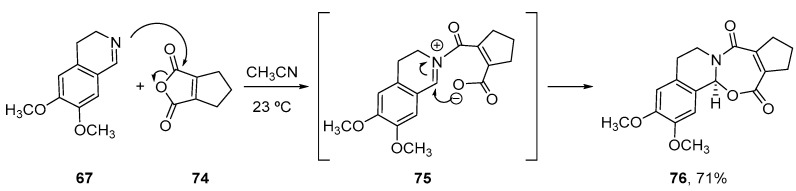
The reaction of cyclopentane-fused maleic anhydride and the possible mechanism.

Surprisingly, anhydride **74** reacts with imines **66**–**68** at room temperature following a different reaction path through intermediate **75** to generate 7-member ring products (**76**; Scheme 26) [36].

**Scheme 27:**
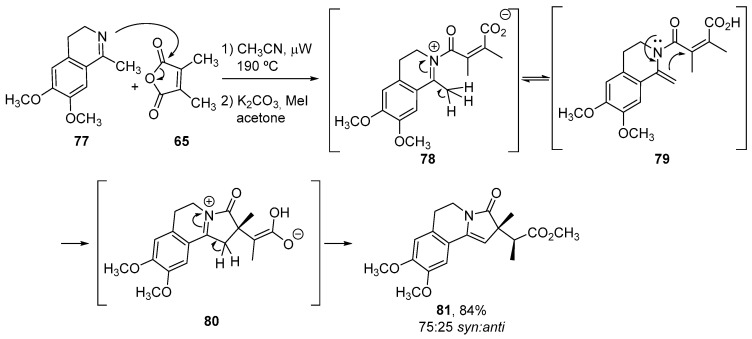
Iminolysis/aza-Michael sequence leading to tricyclic unsaturated lactam **81**.

Similarly, the reaction of disubstituted maleic anhydrides (**65**) with cyclic ketimines (**77**) allowed for obtaining new tricyclic unsaturated lactam CCR adducts (**81**) through an iminolysis/aza-Michael sequence (Scheme 27).

**Scheme 28:**
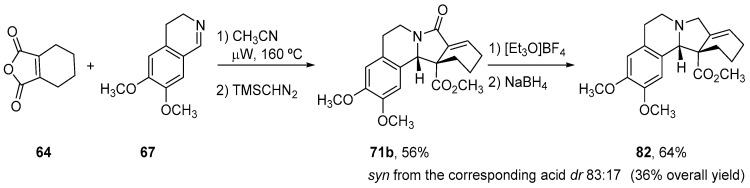
Synthesis of (±)-jamtine **81**.

Interestingly, Royer and co-workers published an identical synthesis of *syn* lactam **71b** simultaneously and independently from that of Shaw. They also reported the reduction of the amide function by treatment with [Et_3_O]BF_4_ followed by NaBH_4_ to yield antihyperglycemic alkaloid jamtine **82** (Scheme 28) [37].

**Scheme 29:**
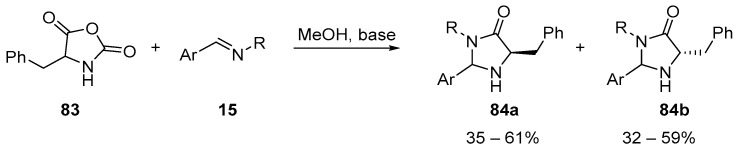
CCR with succinic anhydride analogues containing endocyclic heteroatoms.

Succinic anhydride derivatives in which one of the endocyclic carbons has been replaced with a heteroatom have also been successfully used in CCRs. For instance, Sucu and co-workers employed Leuch´s anhydride (**83**), a special succinic-type anhydride that can be obtained from amino acids, to obtain a CCR-type product **84** by condensation with imines (**15**) in the presence of a base with MeOH as a solvent (Scheme 29) [60].

#### 3.1.2. Six-Membered Cyclic Anhydrides

As part of a work on the development of solvent-free CCRs, Krasavin and colleagues made use of alkyl-substituted glutaric anhydrides, which showed the same behaviour as unsubstituted reagents, to produce the corresponding lactams without significant modifications in yield or diastereoselectivity [35].

**Scheme 30:**
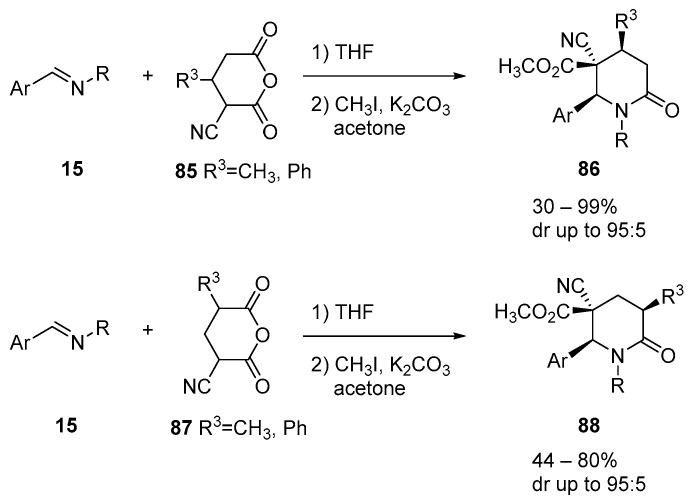
CCR cyano-glutaric anhydrides.

2-Cyano-glutaric anhydrides **85** and **87** have been found to readily react with different *N*-arylarylmethanimines (**15**) to produce 2-piperidinones **86** and **88**, respectively, in high yields. Notably, this process involves the generation of three new stereogenic centres with good diastereoselectivities (Scheme 30) [61].

**Scheme 31:**
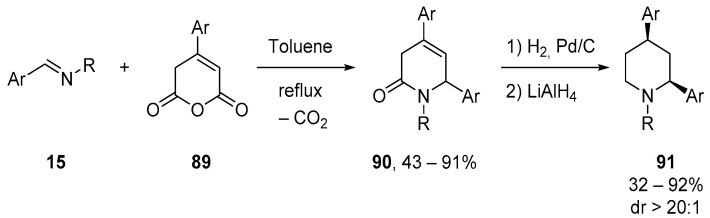
Synthesis of *cis*-2,4-piperidines by a decarboxylative CCR followed by reduction.

Unsaturated 3-arylglutaconic acid anhydrides **89** react with imines to yield the corresponding lactones that decarboxylate in the reaction medium to form 2-piperidinones **90**. Subsequent stereoselective hydrogenation and lactam reduction produce challenging *cis*-configured 1,2,4-trisubstituted piperidines **91** (Scheme 31) [62,63]. In addition, 3-Arylglutaconic acid anhydrides can also react with unprotected oximes to produce the corresponding *N*-hydroxylactams [64].

**Scheme 32:**
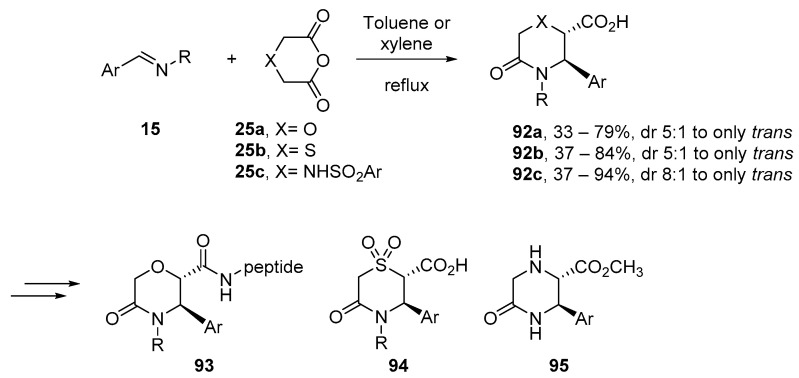
CCR with glutaric anhydride analogues containing endocyclic heteroatoms.

Oxa, aza, and thia analogues of glutaric anhydride react with imines to yield the corresponding δ-lactams [43,65]. For example, the reaction of diglycolic anhydride (1,4-dioxane-2,6-dione, **25a**) with various imines **15** produced 3,4-disubstituted 5-oxomorpholine-2-carboxylic acids **92a**, which were further transformed into peptidic derivatives **93** (Scheme 32) [65]. Analogously, thiodiacetic anhydride **25b**, in similar conditions, produces 5-oxothiomorpholin-2-carboxylic acid products (**92b**) that can be readily oxidised to the corresponding sulfones **94** [43], and, finally, morpholine-2,6-diones **25c** also react satisfactorily to yield piperazine analogues **92c** that can be deprotected to produce **95** [43].

Further, the Vilsmeier–Haack functionalisation of morpholinonates and thiomorpholinonates (**92a,b**) produced persubstituted *N,O*- and *N,S*-heterocyclic vinyl chlorides that were readily coupled with terminal acetylenes and styrenes or subjected to a Wittig/Diels–Alder sequence to generate a variety of heterocyclic scaffolds [66].

**Scheme 33:**
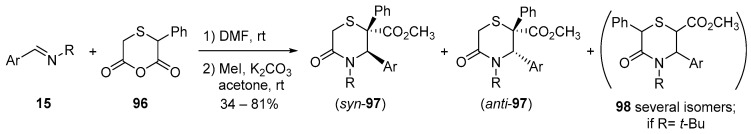
CCR with 3-phenyl-1,4-oxathiane-2,6-dione (**95**).

These hetero-substituted anhydrides (**25a**–**c**) are more prone to enolisation than the parent glutaric anhydride (**19**) and, thus, can react in milder conditions. Aryl substituents in the α position also favour enolisation in this position. The combination of both structural features in 3-phenyl-1,4-oxathiane-2,6-dione (**96**) makes it possible for this anhydride to react at room temperature, yielding a diastereomeric mixture of products (**97**) in an approximate 1:1 ratio (Scheme 33) [67].

Noticeably, the reaction of anhydride **96** with imines containing bulky motifs leads, in addition to the desired *syn/anti* mixture of CCR adducts, to the products of the CCR at the less hindered α-position (**98**), which include three stereogenic centres.

**Scheme 34:**
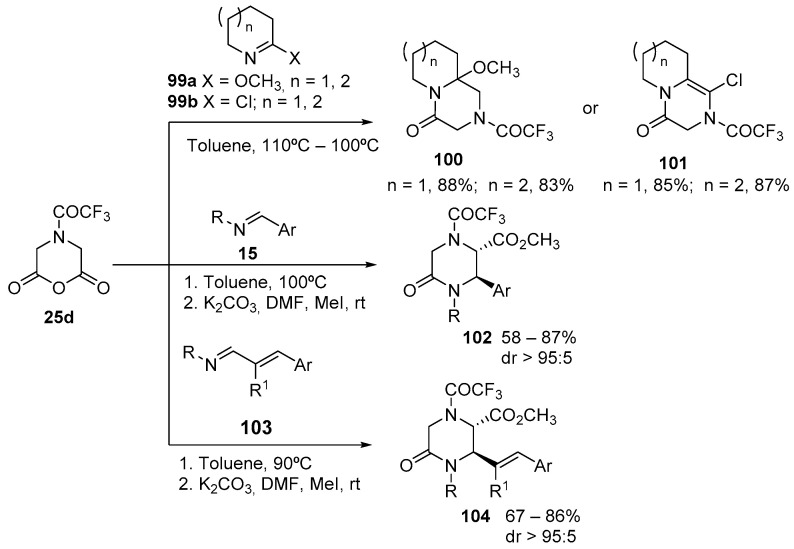
Synthesis of fluorinated ketopiperazines via the CCR.

Recently, the CCR of novel *N*-trifluoroacetyl anhydride (**25d**) produced diastereoselectively fluorinated 3-aryl- (**102**) or 3-styryl- (**104**) ketopiperazines and also bicyclic fluorinated ketopiperazines (**100**, **101**), depending on the starting imine (Scheme 34) [68]. These piperazines could be further transformed into a variety of complex products by means of different post-condensation reactions.

**Scheme 35:**
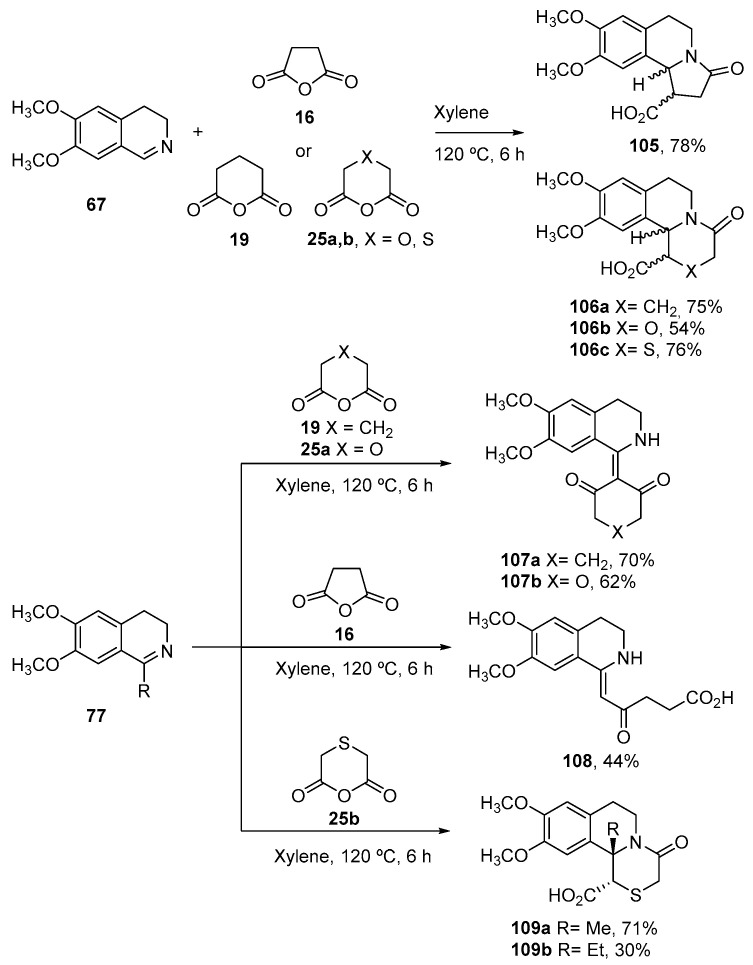
CCR coupling between isoquinoline derivatives and heteroatom-containing anhydrides.

Stanoeva and co-workers reacted succinic (**16**), glutaric (**19**), diglycolic (**25a**) and thiodiacetic anhydrides (**25b**) with isoquinoline (**67**), to obtain tricyclic CCR adducts (**105**, **106**; Scheme 35). Interestingly, analogous reactions of succinic (**16**), glutaric (**19**), or diglycolic (**25a**) anhydrides with 1-substituted isoquinoline (**77**) led to products with exocyclic motifs (**107**, **108**). However, thiodiacetic anhydride (**25b**) showed a different reactivity and produced the expected tricyclic system (**109**) [69].

**Scheme 36:**
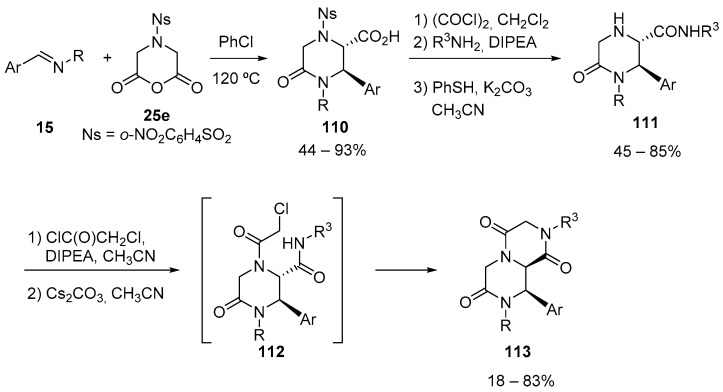
Post-condensation transformation of CCR adducts leading to bicyclic piperazines (DIPEA: *N,N*-diisopropylethylamine).

**Scheme 37:**
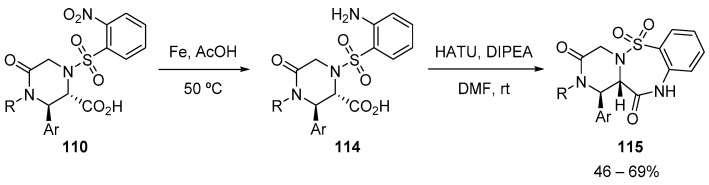
Synthesis of tricyclic sultam-lactam scaffold (DIPEA: *N,N*-diisopropylethylamine, HATU: 2-(7-Aza-1*H*-benzotriazole-1-yl)-1,1,3,3-tetramethyluronium hexafluorophosphate).

Post-condensation transformation of piperazine analogues (**110**) obtained by the CCR of morpholine-2,6-diones (**25e**) affords a straightforward synthesis of bicyclic piperazine peptide β-turn mimetics **113** (Scheme 36) [70]. Additionally, the reduction and lactamisation of piperazine (**110**) yield the unusual 1,2,5-benzothiadiazepin-4-one-1,1-dioxide scaffold (**115**; Scheme 37) [71].

**Scheme 38:**
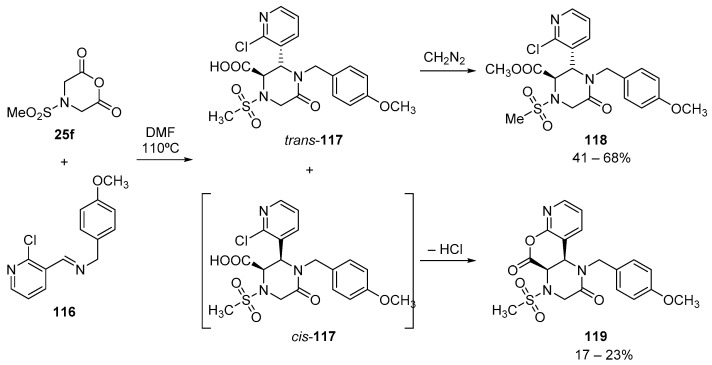
Tricyclic lactone core from CCR couplings.

Analogously, CCR coupling between an imine derivative from 2-chloropyridine-3-carboxaldehyde (**116**) and 4-(methylsulfonyl)-morpholine-2,6-dione (**25f**) led to a mixture of the corresponding *cis*- and *trans*-piperazine derivatives (**117**). The labile chlorine functionality allowed a spontaneous transformation of the *cis-*diastereomers into novel *cis*-tricyclic lactones (**119**; Scheme 38) [72].

**Scheme 39:**
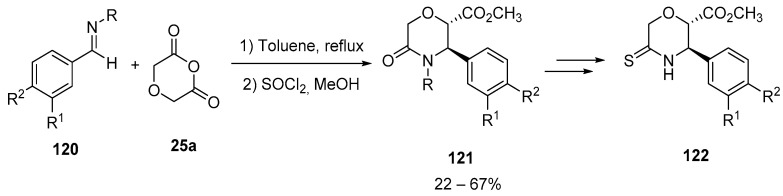
Synthesis of BACE1 inhibitors by the Castagnoli–Cushman reaction.

The condensation of imines (**120**) with diglycolic anhydride (**25a**) and subsequent esterification yields esters (**121**) that can be converted into the corresponding thioamide derivatives (**122**; Scheme 39) which show inhibition activity towards β-secretase 1 (BACE1) [73]. In addition, thioxomorpholino derivatives (**122**) can be further transformed into the corresponding 5-iminomorpholine-2-carboxamides, but these derivatives present less inhibition activity than that of the thioderivatives (**122**).

**Scheme 40:**
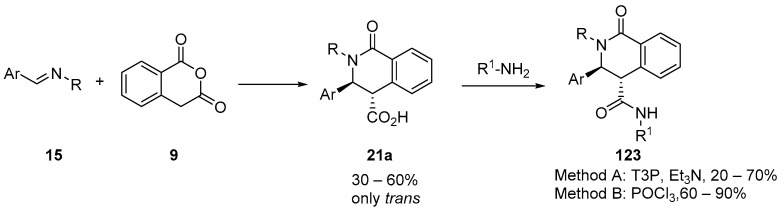
Synthesis of tetrahydro-1-isoquinolone-4-carboxamides (T3P: propanephosphonic acid anhydride).

**Figure 2:**
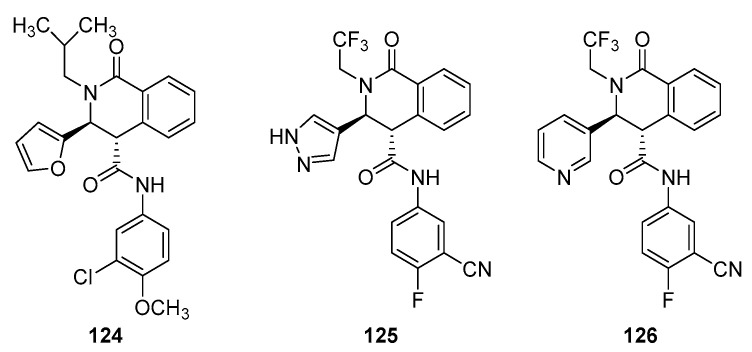
Tetrahydroisoquinolone carboxanilides with antimalarial activity.

Homophthalic anhydride (HPA, **9**) is easily enolisable, and it is one of the most used anhydrides in the CCR. The reaction of different imines **15** with HPA (**9**) affords the racemic *trans* isoquinolones (**21a**) that can be transformed into carboxamide derivatives (**123**) by amide coupling reactions (Scheme 40) [74]. The introduction of different substituents on the N-2, C-3, and C-4 of the tetrahydro-1-isoquinolone-4-carboxanilides (**124**) allows for modulating their physiochemical and pharmacological properties. After investigation of the in vivo antimalarial activity of this series, derivatives **124**–**126** showed high potency, and compound **126** was selected for further study as a preclinical candidate (Figure 2*)* [75].

In a recent application of the CCR, the condensation of DNA-conjugated imines with HPA enabled the synthesis of an isoquinolone core-focused DNA-encoded library [76,77].

**Scheme 41:**
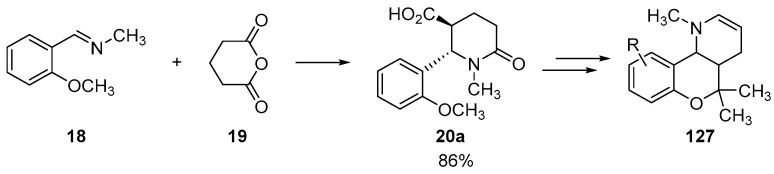
CCR of glutaric anhydride with imines to obtain tetrahydrocannabinol derivatives.

**Scheme 42:**
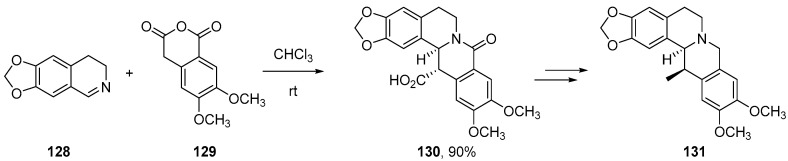
CCR of HPA with imines to obtain polycyclic isoquinolinones.

Castagnoli and Cushman used a 6-membered glutaric anhydride (**19**) and HPA derivative (**129**), respectively, for the preparation of pharmacologically active nitrogen analogues of the tetrahydrocannabinols (**127**; Scheme 41) [26,78] and isoquinolinones, such as tetrahydroprotoberberine alkaloids (**131**; Scheme 42) [27,79]. Stanoeva and co-workers have also performed different transformations on glutaric anhydride (**19**) to yield new substituted piperidin-2-ones [80].

**Scheme 43:**
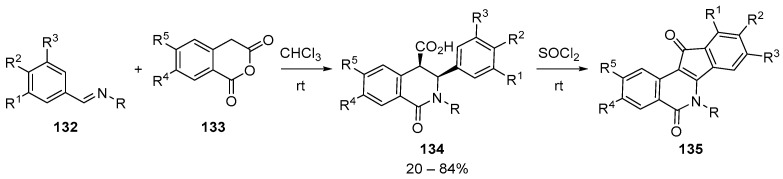
Synthesis of indenoisoquinolines based on a Castagnoli–Cushman reaction.

Cushman and other researchers reacted imines (**132**) and differently substituted homophthalic anhydrides (**133**) to prepare *cis*-substituted isoquinolones (**134**) that were treated with thionyl chloride to yield indenoisoquinolines (**135**; Scheme 43) [81,82,83]. A large number of indenoisoquinolines (**135**) were prepared and screened as inhibitors of topoisomerases and ligands for other biomolecules, providing valuable information to understand and modulate their biological activity [32].

**Scheme 44:**
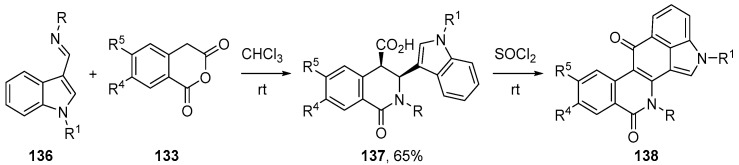
Synthesis of indolophenanthridines.

Indolophenanthridines (**138**) were analogously obtained from homophthalic anhydrides (**133**) and (1*H*-indol-3-yl)methanimines (**136**; Scheme 44) [33].

**Scheme 45:**
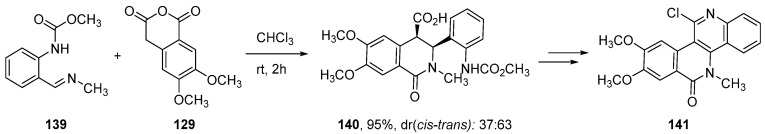
Synthesis of a chloronaphthyridinone topoisomerase I inhibitor.

Other topoisomerase I inhibitors were prepared using the Castagnoli–Cushman reaction as starting point. For example, the reaction of aniline-derived imine (**139**) with 4,5-dimethoxyhomophthalic anhydride (**129**) in chloroform at room temperature affords lactam **140**, which can be further transformed to obtain topoisomerase I inhibitor 11-chloro-8,9-dimethoxy-5-methyldibenzo[*c*,*h*][1,6]naphthyridin-6(5*H*)-one (**141**; Scheme 45) [84,85].

**Scheme 46:**
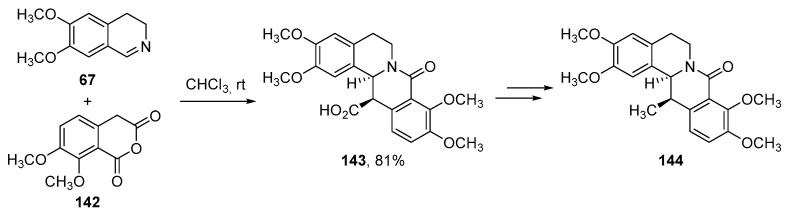
Total synthesis of corydaline based on the Castagnoli–Cushman reaction.

The reaction of 3,4-dimethoxyhomophthalic anhydride (**142**) with the 3,4-dihydro-6,7-dimethoxyisoquinoline (**67**) produced the key intermediate **143** that led to obtaining the alkaloid (±)-corydaline (**144**) in five reaction steps (Scheme 46) [86].

**Scheme 47:**
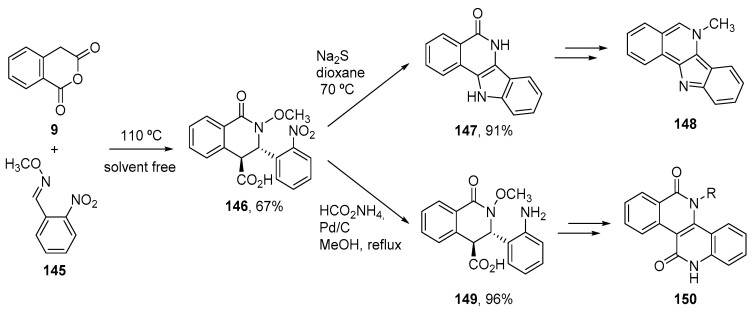
Tetracyclic systems obtained from the Castagnoli–Cushman reaction.

Tetracyclic systems **148** and **150** were prepared by reduction of the Castagnoli–Cushman adduct (**146**) obtained by the reaction of HPA (**9**) with imine **145**. The treatment of 2-methoxy-3-(2-nitrophenyl)-1-oxo-1,2,3,4-tetrahydroisoquinoline-4-carboxylic acid (**146**) with sodium sulfide yields **147**, which is further transformed to indolo[3,2-*c*]isoquinoline (**148**). On the other hand, the reduction of **146** with ammonium formate produces **149** which by cyclisation and dehydrogenation yields dibenzo[*c*,*h*][1,6]naphthyridine (**150**; Scheme 47) [87].

**Scheme 48:**
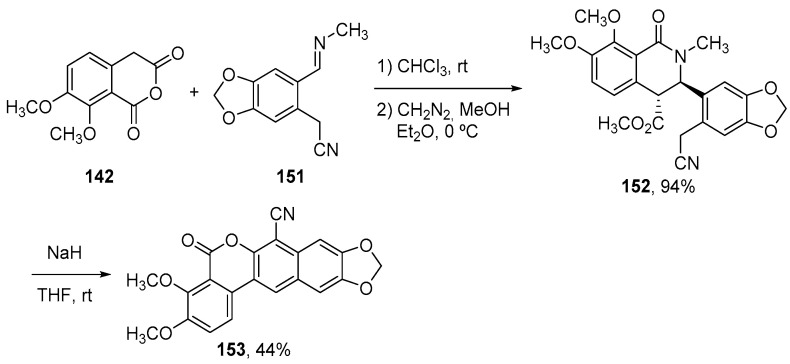
Post-Castagnoli–Cushman transformation leading to angular dibenzopyranes.

Many other post-condensation transformations have been developed by different research groups. For example, the base-triggered rearrangement of the cyanomethyl Castagnoli–Cushman adduct **152** led to 5-benzo[*d*]naphtha[2,3-*b*]pyran (**153**), instead of the expected Dieckmann–Thorpe cyclisation product (Scheme 48) [88].

**Scheme 49:**
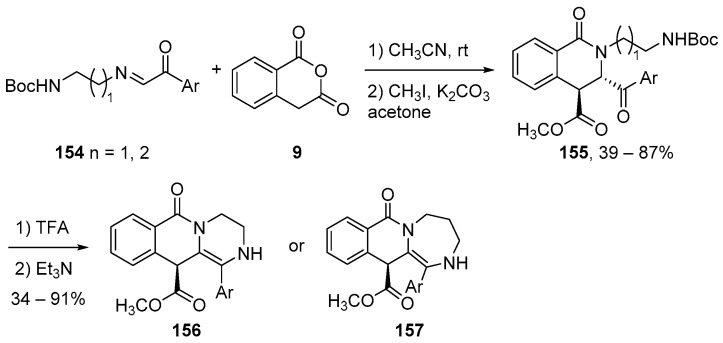
Synthesis of pyrazino- and diazepinoisoquinolones (TFA: trifluoroacetic acid).

The CCR of HPA (**9**) with imines derived from aryl glyoxals and monoprotected diamines (**154**) affords tetrahydroisoquinolines (**155**) that were readily transformed to pyrazino- and diazepino-fused isoquinolones (**156**–**157**) in two chemical operations with moderate or good yields (Scheme 49) [89].

**Scheme 50:**
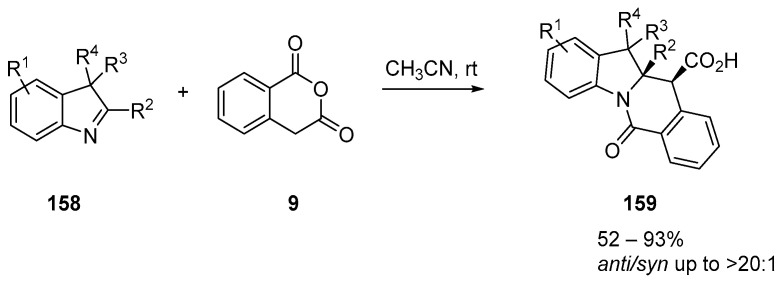
Synthesis of pyrrolo[1,2-*b*]isoquinolines.

Additionally, the reaction of HPA (**9**) with cyclic imine indolenines (**158**) yields natural product-like benzene-fused hexahydropyrrolo[1,2-*b*]isoquinolines (**159**; Scheme 50) [90].

**Scheme 51:**
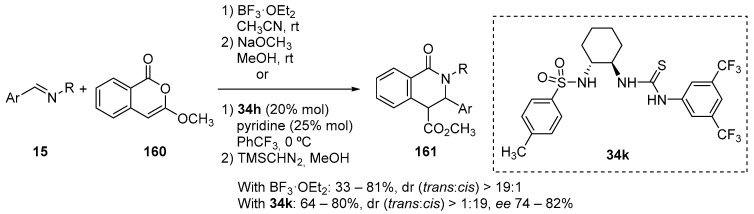
Boron trifluoride catalysed CCR.

As mentioned above, the reaction of imines (**15**) and HPA (**9**) in the presence of chiral thiourea organocatalysts **(34**) was reported to produce the anticipated *cis*-isoquinolonic acids with high diastereo and enantioselectivities (Scheme 13) [50], similarly to what happens with α-aryl succinic anhydrides (Scheme 17) [52]. Seidel and co-workers used alkoxyisocoumarins (**160**) as preformed enolether anhydrides in the presence of boron trifluoride diethyl etherate to yield the corresponding δ-lactams. Treatment with sodium methoxide provoke the epimerisation of the initial *cis/trans* mixtures of products leads to the thermodynamically more stable *trans* isomers (**161**; Scheme 51) [91]. The use of a chiral sulfonamide-thiourea catalyst (**34h**) affords the corresponding lactams (**161**) moderate to good enantioselectivities.

**Scheme 52:**
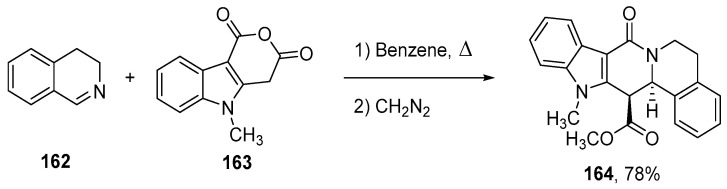
CCR with indole-fused glutaric anhydride.

The reaction of 3,4-dihydroisoquinoline (**162**) with heterohomophthalic anhydrides, such as **163**, yields natural product-like polyheterocycles (**164**; Scheme 52) [92].

**Scheme 53:**
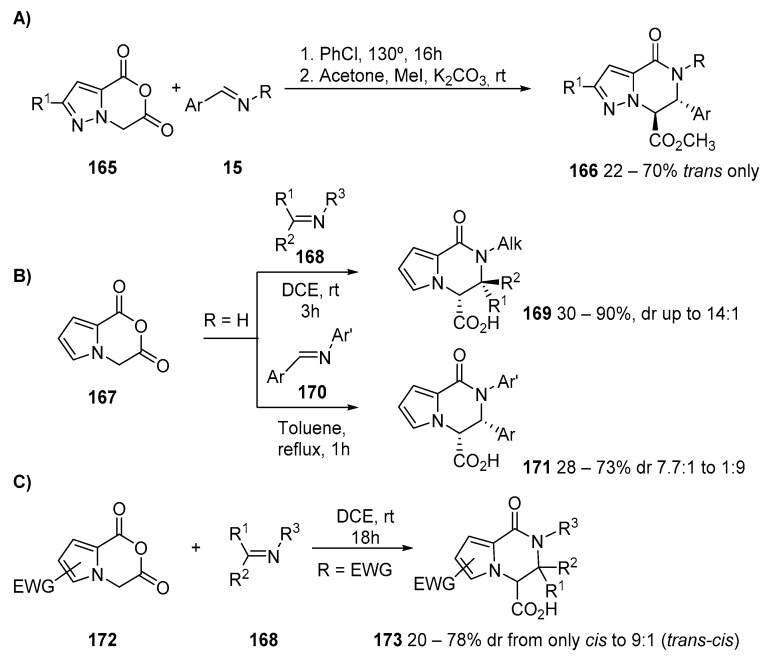
Azole-fused bicyclic anhydrides in CCR (DCE: 1,2-dichloroethane).

Azole-fused bicyclic anhydride derivatives were introduced in cycloaddition reactions by Tamura in 1985 [92], but they have also been exploited in the last decade as promising reagents in the CCR (Scheme 53). Krasavin’s group has made a great effort in this direction. They used (for the first time) a pyrazole-fused cyclic anhydride (**165**) [93] to lead to the corresponding *trans-*CCR adducts (**166**) (Scheme 53A). Moderate 22–70% yields were obtained, possibly due to the formation of several minor by-products. Indole- and benzimidazole-fused anhydrides do not react in the usual CCR conditions; however, the pyrrole bicyclic anhydride **167** [94] showed exceptional features with the CCR (Scheme 53B). This anhydride (**167**) reacts at room temperature with *N*-alkyl- (**168**) and *N*-aryl-imines (**170**) and, remarkably, with “enolisable” α-C-H imines, which is not usual in CCR processes. Remarkably, tetrahydropyrrolopyrazine (**171**) is mostly obtained in its *cis* configuration, except when Ar is a thiophenyl group. Moreover, anhydride **167** readily reacts in the presence of triethylamine with aromatic aldehydes. Krasavin and co-workers argued that this outstanding reactivity can be justified by the efficient resonance stabilisation of its enol form involved in the Mannich-type mechanism. This hypothesis was recently confirmed [95] in a study where some pyrrole bicyclic anhydrides with electron-withdrawing groups (EWG) (**172**) reacted with several imines (**168**), presenting worse results compared to the non-substituted pyrrole-fused bicyclic anhydride (Scheme 53C).

#### 3.1.3. Higher Order Cyclic Anhydrides

**Scheme 54:**
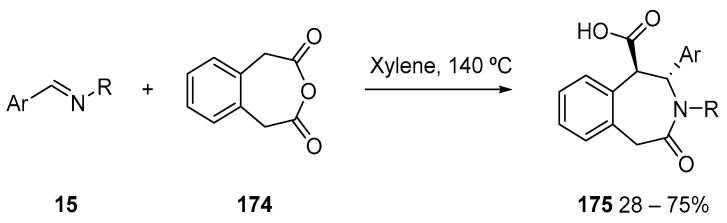
CCR with seven-membered 1,5-dihydrobenzo[*d*]oxepine-2,4-dione.

Higher order anhydrides play a key role in the construction of novel derivatives of CCR. For example, seven-membered cyclic anhydride benzo[*d*]-oxepine-2,4(1*H*,5*H*)-dione (**174**) was found to react with imines (**15**) to afford seven-membered tetrahydrobenzo[*d*]azepine derivatives (**175**) with moderate to good yields and with good *trans* diastereoselectivity (Scheme 54) [96,97].

**Figure 3:**
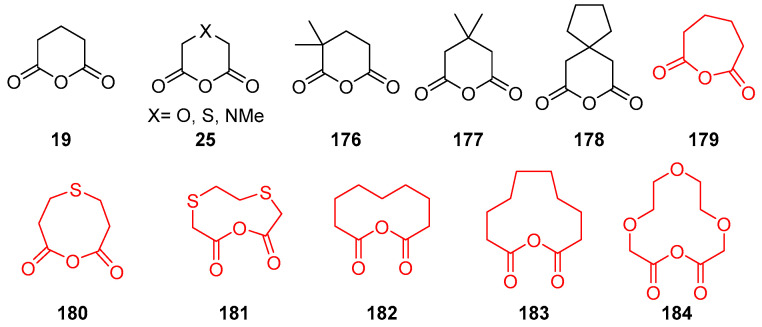
The 6 to 12-membered cyclic anhydrides used in the CCR. Cyclic anhydrides with more than six members are highlighted in red.

**Scheme 55:**
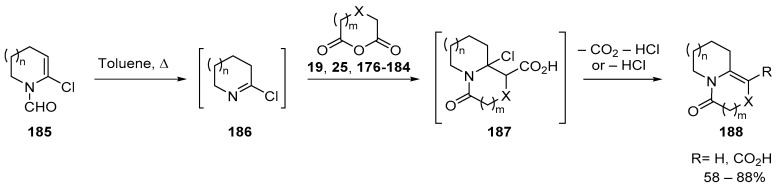
CCR with anhydrides up to 12 members.

Encouraged by these results, Beng and co-workers used a set of cyclic anhydrides of different sizes, up to 12 members, and containing different heteroatoms (Figure 3) in a reaction where the imine is generated in situ by deformylation of cyclic α-chloro eneformamides (**185**; Scheme 55) [98]. This work demonstrates that the Castagnoli–Cushman reaction is not limited to “classic” 5 and 6-membered anhydrides and can provide novel scaffolds in an outstanding way.

**Scheme 56:**
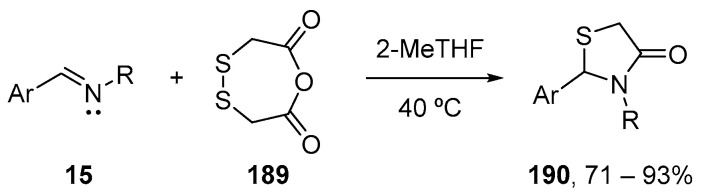
Formal [3+2] cycloaddition of thiodiglycolic anhydride with imines.

Interestingly, Beng and co-workers have recently shown that the thiodiglycolic anhydride (**189**) does not undergo CCR with imines (**15**). Conversely, a formal [3+2] cycloaddition efficiently leads to functionalized 4-thiazolidinones (**190**, Scheme 56) [99].

#### 3.1.4. Diacid Anhydride Precursors

**Scheme 57:**
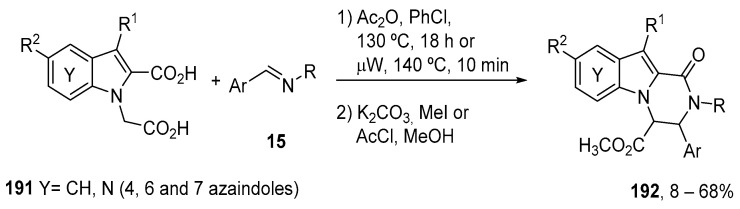
Strategy for the synthesis of tricyclic piperazines.

Imine-anhydride coupling using diacids as anhydride precursors is a valuable strategy for generating CCR adducts that cannot be obtained under conventional conditions [93]. The anhydrides are generated in situ from suitable diacids in the presence of a dehydrating agent and then undergo cycloaddition with the imines. For example, indole diacid **191** was found to react with imines and acetic anhydride to mostly produce the *trans-*isomer of tricyclic piperazines (**192**) in moderate yields (Scheme 57) [100]. In the case of azaindole dicarboxylic acid starting reagents (**191**, Y = N), the reaction was found to be significantly accelerated by microwave irradiation [38].

**Scheme 58:**
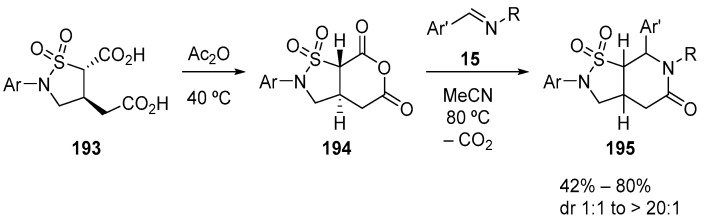
CCR for the synthesis of γ-sultam annelated δ-lactams.

Highly reactive sultam-derived anhydrides (**194**) were obtained by the dehydration of corresponding dicarboxylic acids (**193**) and were reacted in situ with imines (**15**) to yield γ-sultam annelated δ-lactams (**195**) as mixtures of diastereomers (Scheme 58) [101].

**Scheme 59:**
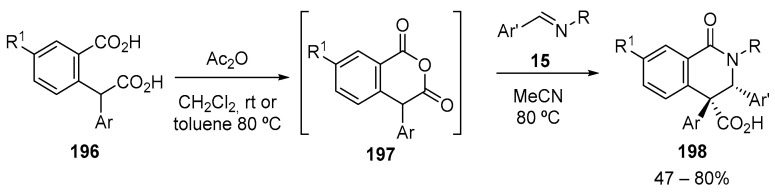
CCR for the synthesis of 4-aryl-substituted tetrahydroisoquinolonic acids.

Krasavin and co-workers also used aryl-substituted homophthalic acids (**196)**, that were dehydrated to the respective anhydrides (**197**) for use in the Castagnoli–Cushman reaction, to yield 4-aryl-substituted tetrahydroisoquinolonic acids (**198**, Scheme 59) [102].

**Scheme 60:**
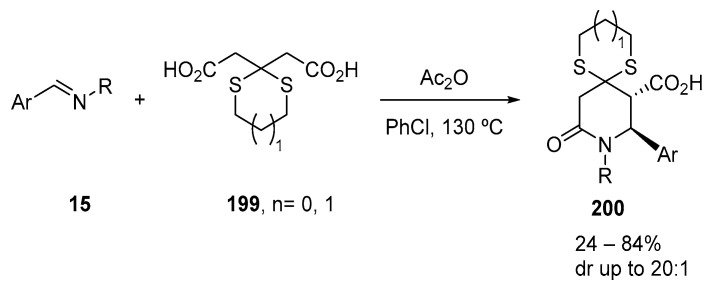
Synthesis of spirocyclic lactams.

Likewise, 1,3-dithiolane and dithiane diacetic acids (**199**) led to spirocyclic lactams (**200**), in generally good yields, through the in situ generated anhydrides (Scheme 60) [103].

**Scheme 61:**
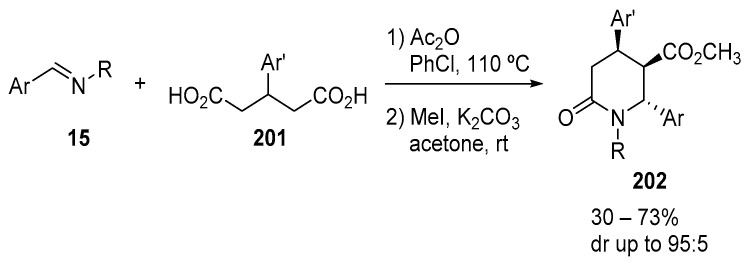
CCR with 3-aryl glutaric acids.

Similarly, *trans*-configured 4,6-diarylpiperidin-2-ones (**202**) with three newly created stereocentres were obtained as predominantly one diastereomer from 3-aryl glutaric acids (**201**; Scheme 61) [104].

**Scheme 62:**
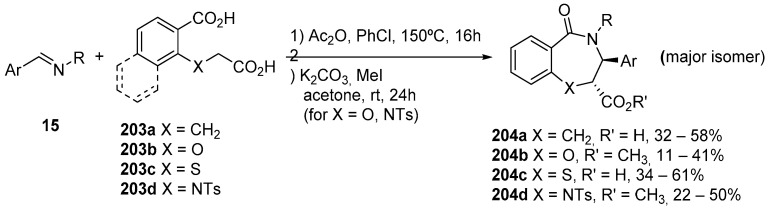
Synthesis of arene-fused seven-membered nitrogen heterocycles.

Diacid precursors of heteroatom-substituted arene-fused seven-membered cyclic anhydrides (**203**) reacted with imines (**15**) in a similar way to yield the corresponding ε-lactams (**204**), in contrast to their poor performance in the anhydride-imine “classic” CCR (Scheme 62) [105].

**Scheme 63:**
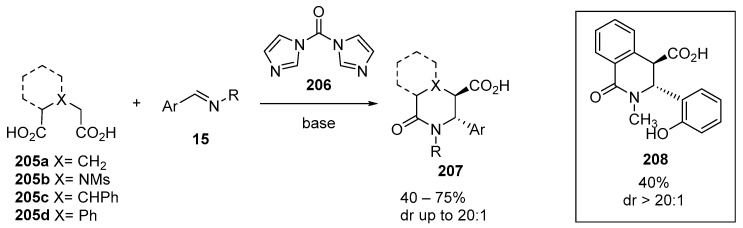
CCR using diacid starting materials in the presence of 1,1′-carbonyldiimidazole.

Transformation of the diacid precursors into the corresponding anhydrides can also be facilitated by other dehydrating agents, such as 1,1′-carbonyldiimidazole (**206**) [106]. This allows for using substrates prone to acylation or sensitive to acidic conditions, such as hydroxyphenyl-substituted imines, that in these conditions afford hydroxyaryl-derived lactams, such as **208** (Scheme 63).

#### 3.1.5. Dicarboxylic Acid Monoesters

**Scheme 64:**
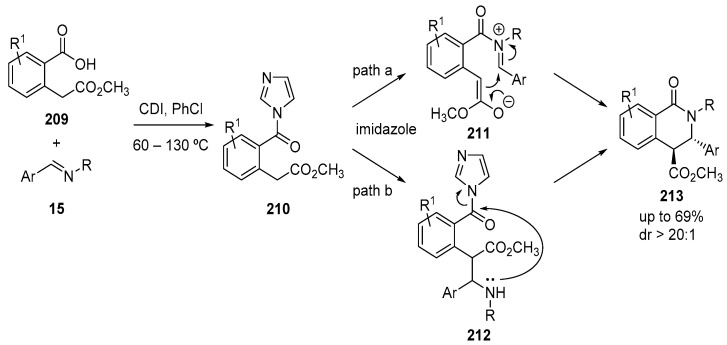
Castagnoli–Cushman-type reaction with homophthalic esters.

Krasavin and co-workers recently reported the use of *o*-(alkoxycarbonyl)methyl benzoic acids (**209**) in place of homophthalic anhydride. Activation of the acid with 1,1′-carbonyldiimidazole (CDI) allows for obtaining tetrahydroisoquinolonic esters (**213**) directly, according to the mechanism shown in Scheme 64 [107].

The scope of this variant of the CCR has been extended to other *o*-methyl benzoic acids α-substituted with electron-withdrawing groups other than an ester. In this way, cyano-, amido-, and sulfonyl-substituted tetrahydroisoquinolones could be readily obtained by activation of the substrate acid with CDI or acetic anhydride [108].

**Scheme 65:**
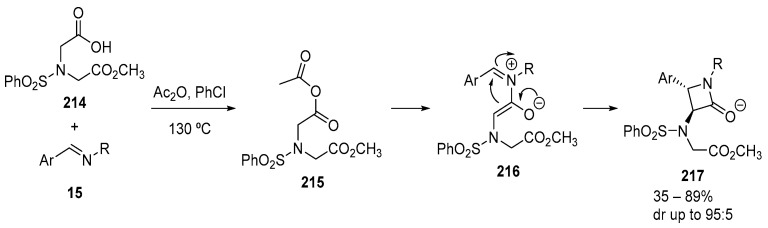
Synthesis of β-lactams from dicarboxylic acid monoesters.

On the other hand, the reaction of *N*-(2-methoxy-2-oxoethyl)-*N*-(phenylsulfonyl)glycine monomethyl esters (**214**) with imines (**15**) promoted by acetic anhydride led to β-lactams (**217**), instead of the initially expected Castagnoli–Cushman-type δ-lactams (Scheme 65) [109]. The products were obtained by a cyclisation involving a methylene group adjacent to the acid moiety.

### 3.2. Scope of the Imine Substrates

**Scheme 66:**
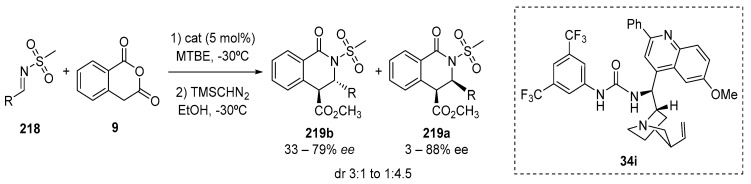
CCR with *N*-sulfonylimines.

**Scheme 67:**
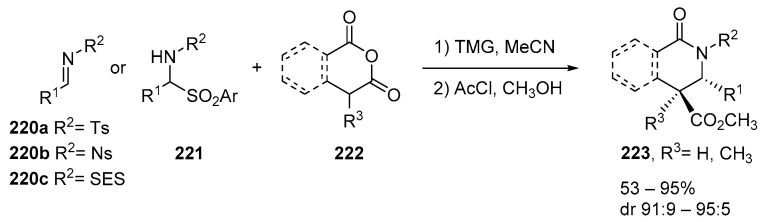
CCR with *N*-sulfonylimines and aminosulfones (TMG: 1,1,3,3-tetramethylguanidine, SES: 2-(trimethylsilyl)ethanesulfonyl).

Continuing the exploration of incorporating new reagents in the CCR, the imine component has also received recent attention. Thus, some research groups have introduced new functionalities, such as sulphur-containing groups. For instance, Cronin and co-workers reacted *N-*mesyl imines (**218**) and HPA (**9**) in the presence of asymmetric catalyst **34i** in Methyl *tert*-butyl ether (MTBE) to afford the corresponding lactams (**219**). These were obtained in good yields with a preponderance of the *trans*-isomer (**219b**) in most of the cases (Scheme 66) [51]. Shaw also reported that *N-*sulfonyl imines (**220**), or the corresponding precursor aminosulfones (**221**), produced the desired CCR adducts (**223**) in high yields and good diastereoselectivity in a base-catalysed process with different anhydrides (**222**; Scheme 67) [110]. A similar strategy was also followed by Beng [98]. Notably, as already mentioned, a reaction with *N*-sulfonylimines was the key step in the synthesis of bisavenanthramide B-6 (**62**), one of the most successful applications of the CCR to total synthesis (Scheme 22) [57].

**Scheme 68:**
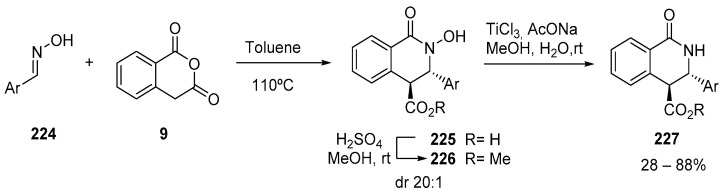
CCR with hydroxylamine and a post-condensation reduction.

Hydroxylamines (**224**) have also been employed as nitrogen sources for CCR to yield NH-tetrahydroisoquinolonic derivatives (**227**; Scheme 68) [111]. Bakulina and co-workers used this strategy in a synthesis of siderophores [112,113,114].

**Scheme 69:**
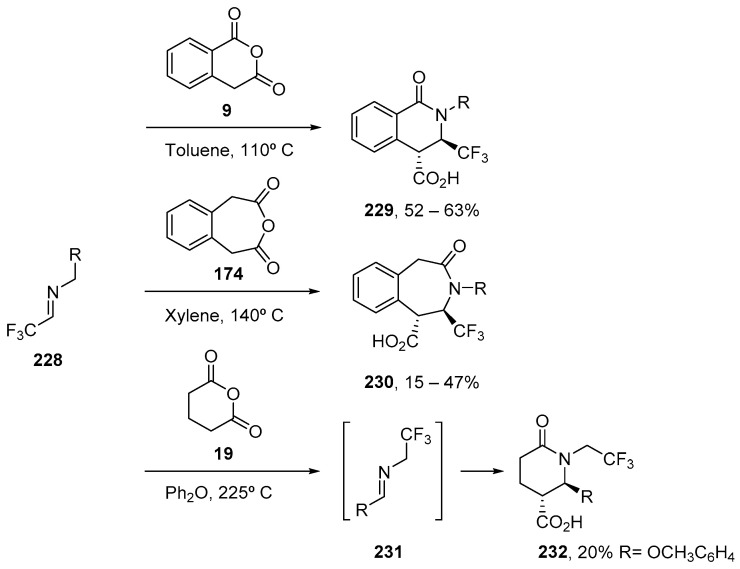
Synthesis of fluorinated CCR adducts.

Notably, in a recent study, fluoral hydrate was revealed to be a suitable precursor for trifluoromethylimines (**228**), which can react with several anhydrides in CCRs despite the significant decrease in reactivity due to the electron-withdrawing effect of the trifluoromethyl group (Scheme 69) [115]. However, only HPA (**9**) and 1,5-dihydrobenzo[*d*]oxepine-2,4-dione (**174**) were shown to react with trifluoromethyl imines (**228**) in an expected manner to yield, respectively, trifluoro-substituted lactams **229** and **230**. Glutaric anhydrides (**19**) require harsh reaction conditions and the lactams obtained (**232**) result from the CCR of isomeric trifluoromethyl imines (**231**) formed by a [1,3]-hydride shift under thermal conditions. In contrast, succinic and succinic-derived anhydrides did not lead to any additional products.

**Scheme 70:**
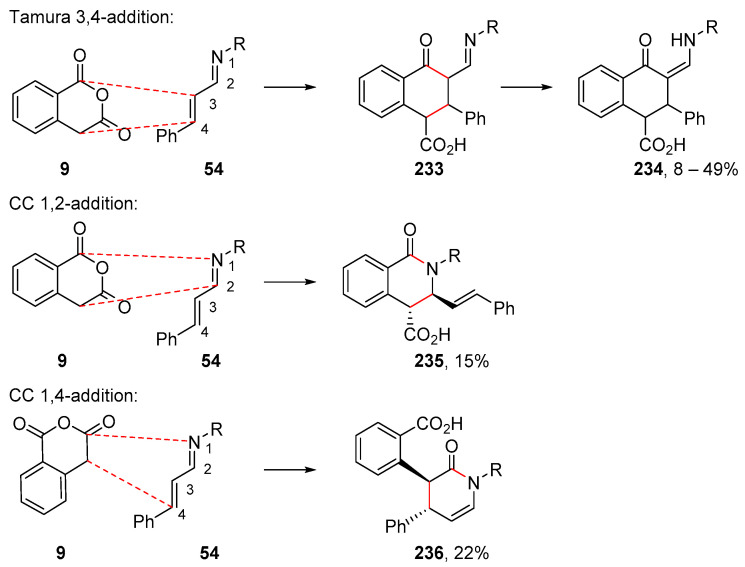
Possible pathways for the reaction of 1,3-azadienes with HPA leading to [4+2] cyclisations.

Haimova, De Kimpe, and co-workers found that cinnamaldimines (**54**) react with HPA (**9**), affording the diastereoisomeric naphthalenones (**234**) along with 3,4-dihydro-l(2*H*)-isoquinolinone-4-carboxylic acids (**235**) and 3,4-dihydro-l(2*H*)-pyridinone (**236**). The major product (**234**) is produced by a Tamura 3,4-addition, while 1,2- and 1,4- Castagnoli–Cushman additions give rise, respectively, to compounds **235** and **236** (Scheme 70) [116,117].

**Scheme 71:**
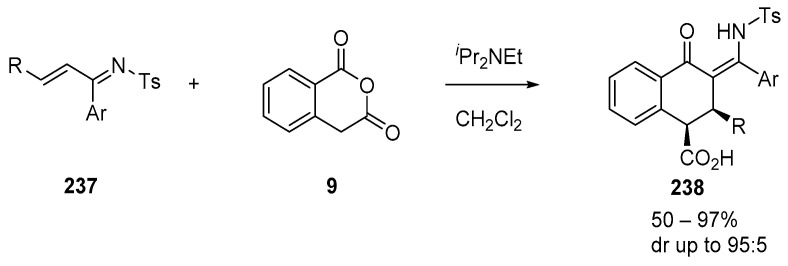
Tamura reaction of HPA with conjugated ketoimines.

Later work by Shaw and co-workers demonstrated that conjugated ketoimines containing a tosyl group on the nitrogen (**237**) can react with enolisable anhydrides, such as HPA (**9**), in the presence of a base through the C-C double bond via a Tamura reaction (Scheme 71) [118].

**Scheme 72:**
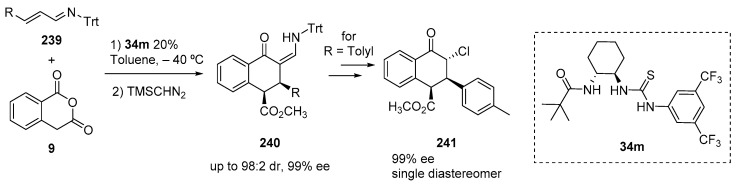
CCR for the synthesis of vinylogous amides and α-chlorotetralones.

The reaction of α,β-unsaturated *N*-trityl imines (**239**) with HPA (**9**) in the presence of anion-binding bifunctional catalysts (**34m**) allows for the synthesis of vinylogous amides (**240**) with high enantio- and diastereocontrol. Compound **240** can be easily transformed into α-chlorotetralone (**241**) by treatment with *N*-chlorosuccinimide (NCS) through the corresponding α-chloroimine, which undergoes a hydrolysis-oxidation-β-ketodecarboxylation cascade (Scheme 72) [119].

**Scheme 73:**
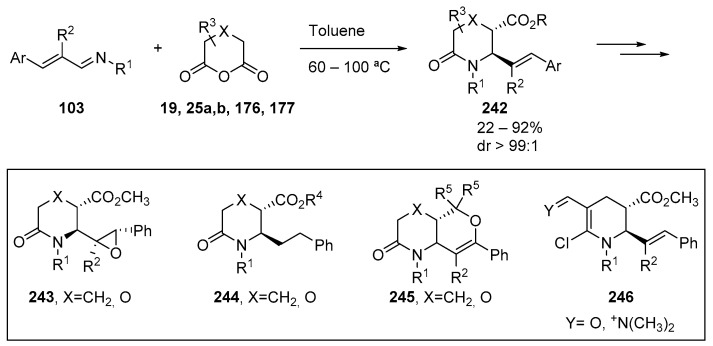
The reaction of 1,3-azadienes with hexacyclic anhydrides and their post-condensation products.

Furthermore, the reaction of hexacyclic anhydrides (**19**, **25**, **176** or **177**) with α,β-unsaturated imines (**103**) results in the formation of allylic lactams (**226**), which can undergo a variety of reactions to produce diverse scaffolds. For example, they can undergo epoxidation to yield epoxylactams (**243**), catalytic hydrogenation to afford (**244**), Pd-catalysed dehydrogenative cross-coupling to obtain azabiclycles (**245**), or regioselective Vilsmeier-Haack reaction to produce α-chloro-β-formyl dihydropiperidine (**246**; Scheme 73) [120].

*N*-iodoaryl-substituted allylic lactams synthesised in this fashion have been transformed into *N*-stilbeno or styrenyl piperazine derivatives by CuBr-catalysed alkenylation with simple alkenes [121].

**Scheme 74:**
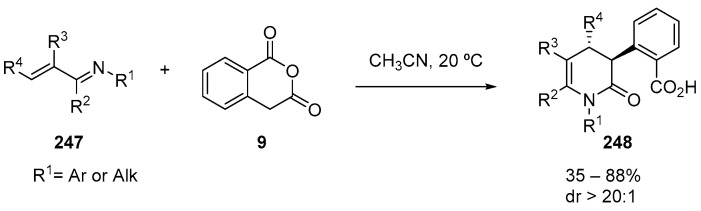
Castagnoli–Cushman-type 1,4-addition of conjugate imines to HPA.

The reaction of chalcone imines (**247**) and HPAs (**9**) by a 1,4-imine addition in a process related to the CCR has also been recently described (Scheme 74) [122]. This reaction yields dihydropyridin-2(1*H*)-ones (**248**) through the nucleophilic addition of HPA enolate to the C-4 position of the 1-aza-1,3-diene followed by intramolecular *N*-acylation, in accordance with the mechanism previously proposed by Haimova and De Kimpe (Scheme 70) [116,117].

**Scheme 75:**
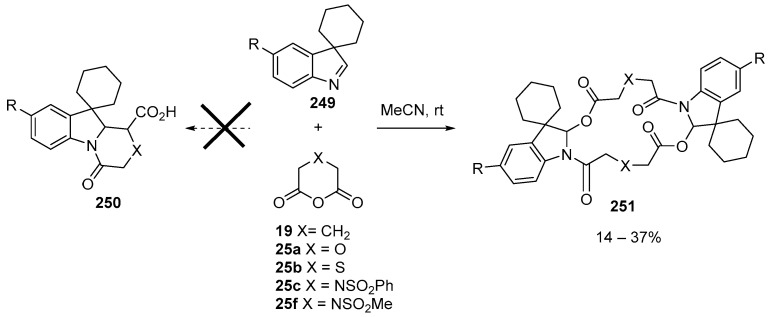
CCR with indolenines.

It is also worth mentioning the reactions of cyclic anhydrides with “less conventional” nitrogen compounds. For example, the reaction between sterically hindered cyclic imines, such as indolenines (**249**), and different substituted glutaric anhydrides (**19** or **25**) leads to a non-expected 16-membered macrocycle (**251**), instead the expected lactam (**250**) [123] (Scheme 75).

**Scheme 76:**
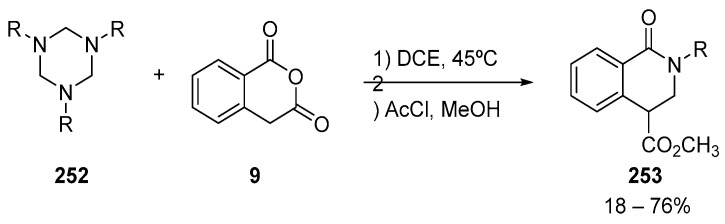
CCR with 1,3,5-triazines.

Moreover, a novel 3-unsubstituted tetrahydroisoquinolonic acid scaffold (**253**) could be achieved in 18–76% yields by the cycloaddition of 1,3,5-triazines (**252**) and HPA (**9**; Scheme 76) [124].

**Scheme 77:**
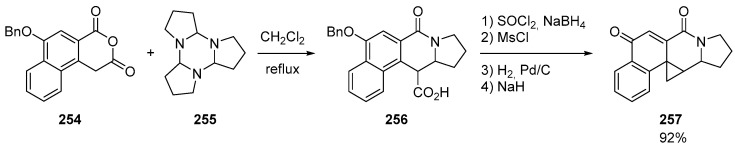
Synthesis of a duocarmycin analogue through CCR/post-condensation.

A similar 1-pyrroline trimer (**255**) was used by Smith and co-workers to produce a tetracyclic Castagnoli–Cushman adduct (**256**) that was subsequently transformed into a doucarmycin analogue (**257**; Scheme 77) [125].

**Scheme 78:**
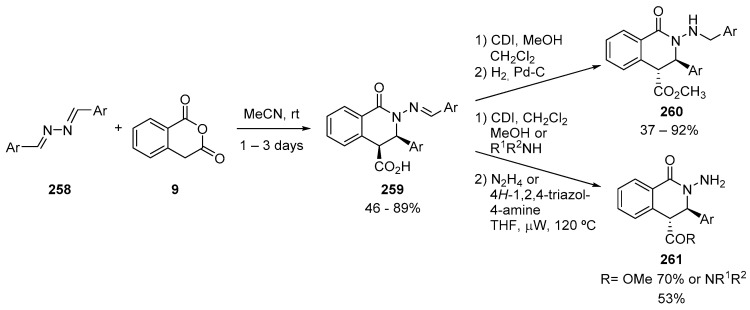
CCR with aldazines (CDI: 1,1’-carbonyldiimidazole).

Finally, aldazines (**258**) are also suitable reagents for CCR with HPA (**9**) [126]. The reaction takes place in MeCN and produces the corresponding *cis*-carboxylic acids (**259**), which can be further methylated to yield the corresponding *trans*-diastereomer esters (**260**; Scheme 78). This approach has been used in the effective synthesis of *N*-aminolactams (**261**).

**Scheme 79:**
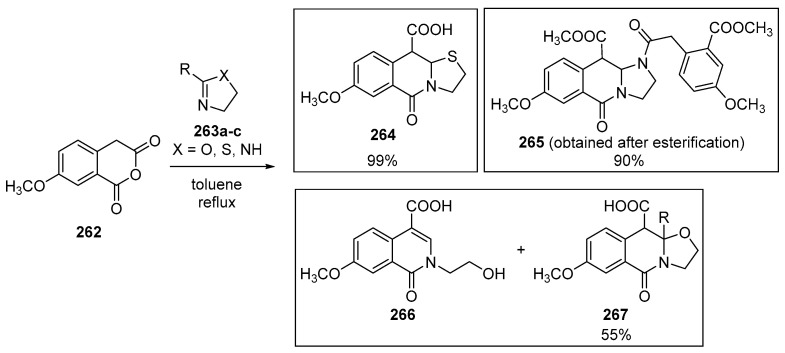
Oxazolines, thiazolines, and imidazolines in CCR.

The use of cyclic imines, especially 3,4-dihydroisoquinolines (**162**) [27,36,69,78,79,80,86,90,92] and precursor cyclic α-chloro eneformamides (**185**) [98], in CCRs has been mentioned above. Other heterocyclic imines, such as oxazolines, thiazolines, and imidazolines (**263a**–**c**), were introduced by Smith *(*Scheme 79) [127] and Palamareva [128,129].

**Scheme 80:**
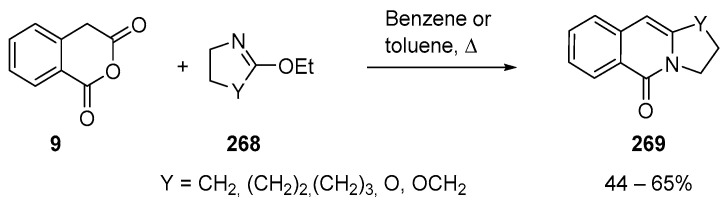
Cyclic imino ethers in the CCR (from [130]).

**Scheme 81:**
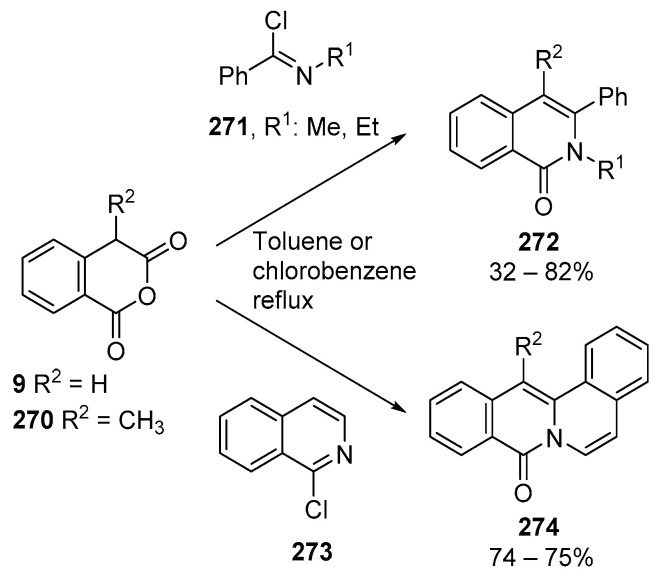
Imidoyl chlorides in the CCR (from [131,133]).

Earlier, Copola [130] and Haimova [131,132,133] made use of imino ethers (**268**; Scheme 80) and imidoyl chlorides (**271**, **273**; Scheme 81) in the CCR with homophthalic anhydrides (**9**).

## 4. Three-Component Castagnoli–Cushman Reactions (3C-CCRs)

### 4.1. Limitations

**Scheme 82:**
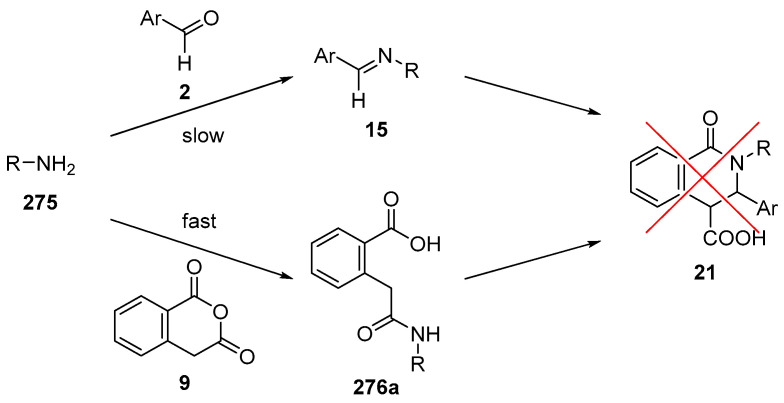
Chemical transformations when mixing an anhydride, an amine, and an aldehyde, which supposedly hinder the success of a 3C-CCR.

Although only an imine (**15**) and an anhydride (**9**) participate in the classical Castagnoli–Cushman reaction, this has usually been considered a multicomponent reaction, since the imine (**15**) can be obtained from the corresponding amine (**275**) and aldehyde (**2**). However, until recently it was assumed that anhydrides (**9**) cannot participate in real three-component reactions with aldehydes (**2**) and amines (**275**). It was commonly believed that the amine (**275**) rapidly reacts with the less hindered anhydride carbonyl to irreversibly yield a monoamide (**276a**) that can no longer incorporate the aldehyde component (Scheme 82). However, there is recent evidence that the reaction of the amine (**275**) with the anhydride (**9**) is reversible under certain conditions, making the coexistence of anhydride (**9**) and imine (**15**) in the reaction medium possible to some extent.

In the last two decades, different solutions have been adopted to perform the CCR in a multicomponent manner. These are discussed in the following sections.

### 4.2. Sequential Reactions

The reaction of an aldehyde and an amine to yield a corresponding imine can be combined sequentially with the subsequent reaction with an anhydride. Ryabukhin and co-workers have used this simple strategy to combinatorially synthesise a library of 132 1,2-disubstituted 5-oxopyrrolidine- and 6-oxopiperidine-3-carboxylic acids [134].

Analogously, a diacid and a dehydrating agent, such as acetic anhydride can be added to the recently formed imine with a similar result [135].

### 4.3. Catalysed and Thermal 3C-CCRs

**Scheme 83:**
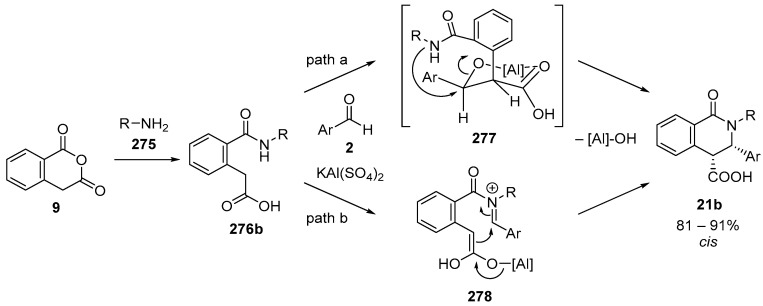
The mechanism proposed by Azizian for the 3C-CCR catalysed by KAl(SO_4_)_2_·12 H_2_O.

Remarkably, the use of catalysts not only performs the reaction more efficiently and in milder conditions, but also permits, in certain cases, carrying out the three-component reaction with HPA (**9**), aldehydes (**2**), and amines (**275**). Azizian and co-workers reported that KAl(SO_4_)_2_·12 H_2_O (alum) effectively catalyses this reaction to yield *cis*-isoquinolonic acids (**21b**) with complete stereoselectivity (Scheme 83) [136]. The authors reported the isolation of a supposed intermediate amido acid (**276b**) that they argue would be transformed into the desired product (**21b**) when the reaction time was further extended. According to these results, they proposed a reaction mechanism via the initial attack of the amine (**275**) to HPA (**9**) to yield intermediate **276b**. The carboxylic acid enolate on this intermediate would react with the aldehyde (**2**) in the presence of alum through a cyclic six-membered Zimmerman–Traxler transition state to yield intermediate **277** (path a); the intramolecular nucleophilic attack of the amide nitrogen with HO-Alum elimination would yield the final product (**21b**). An alternative mechanism for the transformation of intermediate **276b** into the final product (path b) would involve the reaction of the amide nitrogen with the aldehyde to form an intermediate *N*-acyliminium cation (**278**) and a subsequent cyclisation with the acid enolate. According to the authors, the catalyst is necessary to allow the transformation of intermediate **276b**, which in its absence would remain stable, hampering the three-component reaction.

**Scheme 84:**
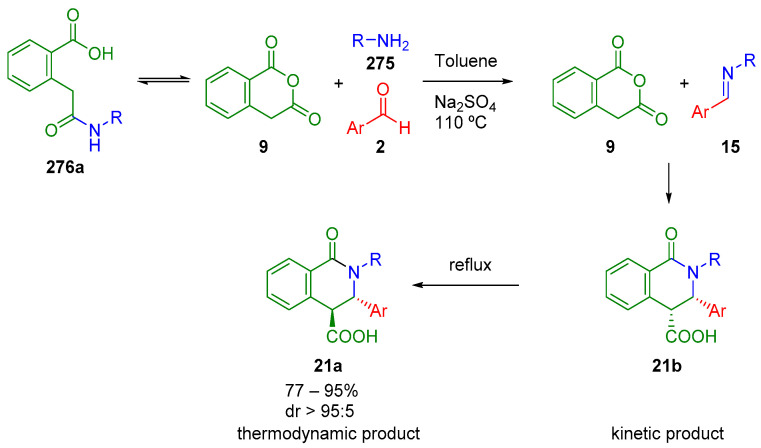
The mechanism for the 3C-CCR proposed by Shaw.

However, recent evidence found by Krasavin [137] and Shaw [59] indicate that the reaction of HPA (**9**) with amines (**275**) actually yields the amido acid isomer **276a** and not **276b**. Based on solid mechanistic investigations, Shaw proposed a mechanism for 3C-CCR in which cyclic anhydride (**9**) and amido acid (**276a**) are in equilibrium. In this scenario, the amine (**275**) and aldehyde (**2**) would form an imine (**15**) that would further react with the anhydride (**9**) in a similar way to what occurs in the 2C-CCRs (Scheme 84) [59].

Other heterogeneous Lewis acid catalysts, such as silica/sulfuric acid [138], ZnCl_2_, AlCl_3_-SiO_2_ [139], and sulfonic acid functionalised silica [140] were found to analogously catalyse the reaction of amines (**275**), aldehydes (**2**), and HPA (**9**) to diastereoselectively afford the corresponding *cis*-isoquinolonic acids (**21b**). Similarly, the three-component reaction takes place smoothly in ionic liquids to also yield the *cis* isomers [141]. Other Lewis acids, such as ytterbium(III) triflate [142] and iodine[143] have also been shown to be very convenient catalysts, allowing the reaction to take place in CH_2_Cl_2_ at room temperatures to afford again the corresponding kinetic *cis*-isoquinolonic acid derivatives (**21b**) with high diastereoselectivity. Importantly, the reaction catalysed by ytterbium(III) triflate [142] has been successfully performed also with enolisable aliphatic imines, suggesting that it might take place through a mechanism different from the uncatalysed reactions. Yu and co-workers have also described the 3C-CCR of HPA (**9**), amines (**275**), and aliphatic aldehydes in the presence of excess trimethyl orthoformate [144].

The use of organocatalysts, such as *L*-proline, also succeeded in promoting the reaction of aromatic aldehydes (**2**), anilines (**275**, R = Ar), and HPA (**9**) [145] to produce *cis*-isomers.

**Scheme 85:**
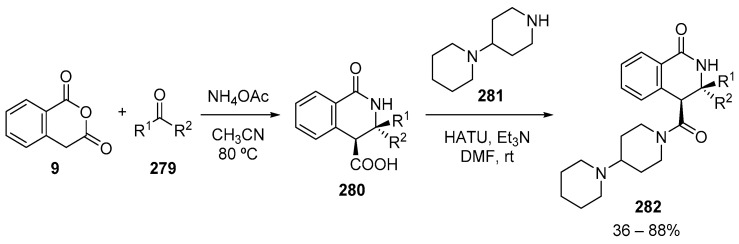
3C-CCR used in the synthesis of PARP inhibitors (HATU: 2-(7-Aza-1*H*-benzotriazole-1-yl)-1,1,3,3-tetramethyluronium hexafluorophosphate).

In contrast with other catalysed CCRs, the two-component reaction of imines (**15**) with HPA (**9**) catalysed by excess BF_3_-Et_2_O leads to *trans*-isoquinolonic acids (**21a**) with a high stereoselectivity [146]. In addition, the three-component combination of HPA (**9**), aldehydes (**2**), and ammonium acetate in the presence of 50% molar aspartic acid as an organocatalyst affords *trans*-isoquinolonic acids (**21a**) [147]. Remarkably, a catalyst-free reaction of HPA (**9**), ammonium acetate, and carbonyl compounds (**279**) was recently reported by Krasavin [148]. It is worth noting that these reactions are performed in refluxing CH_3_CN, which could explain the formation of the thermodynamically more stable *trans* isomers (**280**). This protocol applies to cyclic ketones, leading to spirocyclic lactams [148,149], and has also been used for the preparation of poly(ADP-ribose) polymerase (PARP) inhibitors [150] (**282**; Scheme 85). In agreement with this, Shaw has also demonstrated the feasibility of the 3C-CCR of HPA (**9**), aldehydes (**2**), and both aliphatic and aromatic amines (**275**) without the necessity of a catalyst [59]. This supports his mechanistic proposal (Scheme 84) and situates the Castagnoli–Cushman reaction as a real multicomponent reaction.

### 4.4. 3C-CCR with Diacids

**Scheme 86:**
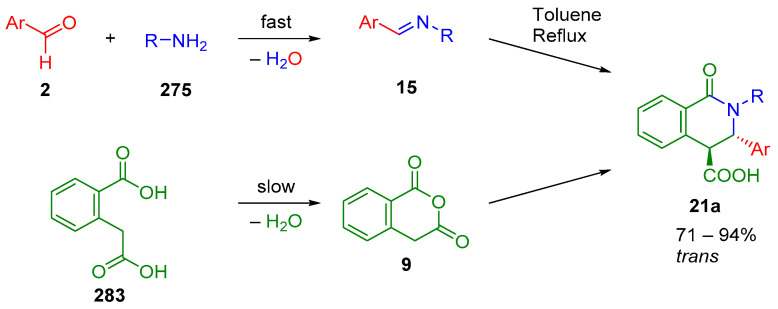
CCR from diacids.

To avoid the formerly assumed irreversible attack of the amine on the anhydride in three-component uncatalysed CCRs, it has been proposed that this can be generated in situ from the corresponding diacids. Unfortunately, the use of dehydrating agents, such as acetic anhydride [135] or 1,1′-carbonyldiimidazole [106], is not compatible with the presence of amines. However, Krasavin showed that the three-component reaction of homophthalic acid (**283**), amines (**275**), and aldehydes (**2**) proceeds satisfactorily with the simultaneous azeotropic removal of water. Under these reaction conditions, rapid formation of the imine (**15**) occurs before HPA (**9**) can be generated from the acid (**283**; Scheme 86) [137].

**Scheme 87:**
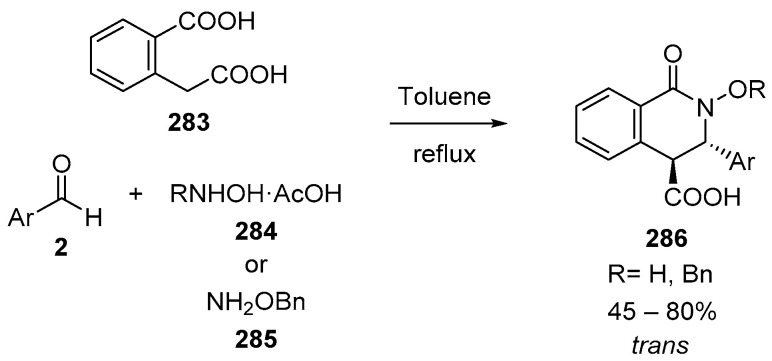
Synthesis of cyclic hydroxamic acids.

Cyclic hydroxamic acids (**286**) were likewise prepared using hydroxylamine acetate (**284**) or *O*-benzylhydroxylamine (**285**) in place of the usual amines (Scheme 87) [151].

**Scheme 88:**
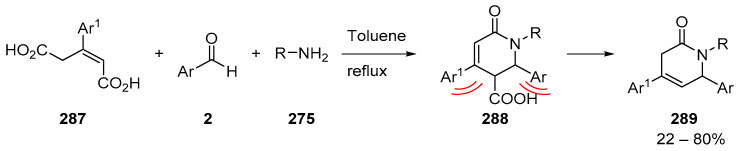
CCR of 3-arylglutaconic acids.

Furthermore, 3C-CCR of 3-arylglutaconic acids (**287**) with aromatic aldehydes (**2**) and amines (**275**) results in sterically congested (as shown by the red coloured arcs) Castagnoli–Cushman adducts (**288**) that spontaneously decarboxylate to afford 4,6-diaryl-1,6-dihydropyridin-2(3*H*)-ones (**289**; Scheme 88) [62,152].

## 5. Four-Component Castagnoli–Cushman Reactions (4C-CCRs)

**Scheme 89:**
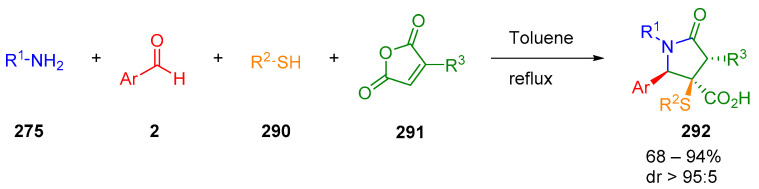
Four-component CCR with maleic anhydrides and thiols.

A four-component version of the CCR, starting from amines (**275**), non-enolisable aldehydes (**2**), maleic anhydrides (**291**), and thiols (**290**), affords, in a single step, γ-lactams (**292**) with up to three new stereogenic centres with high diastereoselectivity (Scheme 89) [42].

This approach is also useful for the preparation of *N*-unsubstituted γ-lactams, which can be further functionalised through acylation and arylation. For this purpose, different sources and synthetic equivalents of ammonia have been used [153].

**Scheme 90:**
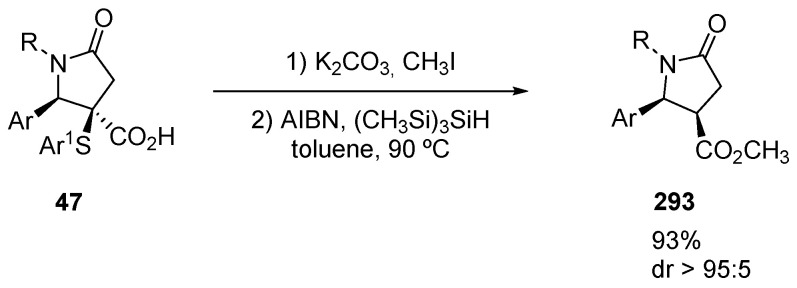
Synthesis of 4,5-disubstituted-2-pyrrolidinones by the desulfurisation of CCR adducts (AIBN: 2,2’-azobis(isobutyronitrile)).

The desulfurisation of γ-lactams (**292**, **47**) leads to the otherwise difficult to obtain corresponding *cis*-4,5-disubstituted-2-pyrrolidinones (**293**; Scheme 90) [42].

**Scheme 91:**
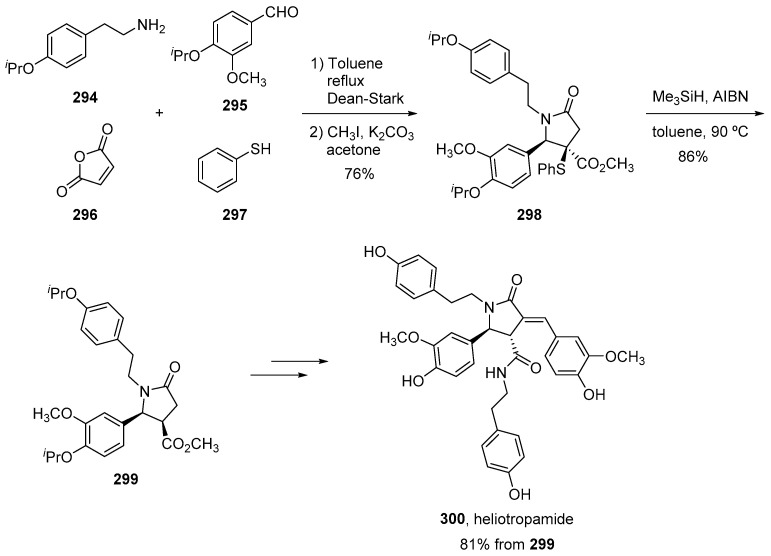
Synthesis of heliotropamide based on the CCR.

Further transformation of the thioether group in sulfur-substituted γ-lactams increases the attainable molecular diversity and has been applied to the synthesis of several natural product analogues. For example, 4C-CCR followed by reductive desulfurisation, amidation, and aldol condensation leads to the natural product heliotropamide (**300**; Scheme 91) [154].

**Figure 4:**
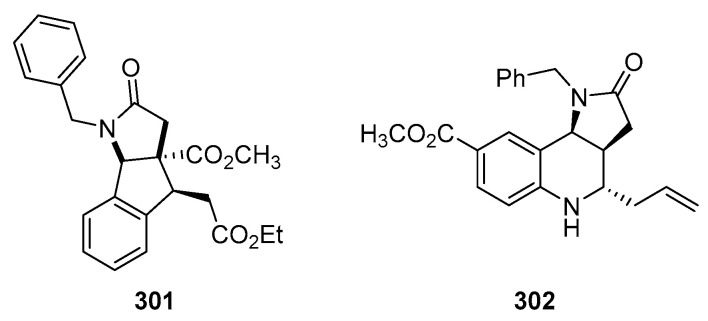
Some natural product derivatives obtained by the transformation of sulphur substituted CCR adducts.

Other natural-like products, such as aza-triquinane (**301**) [155] and a martinellic acid analogue (**302**) [55], could be obtained using similar strategies (Figure 4).

## 6. Reactions with Isatoic Anhydride

**Scheme 92:**
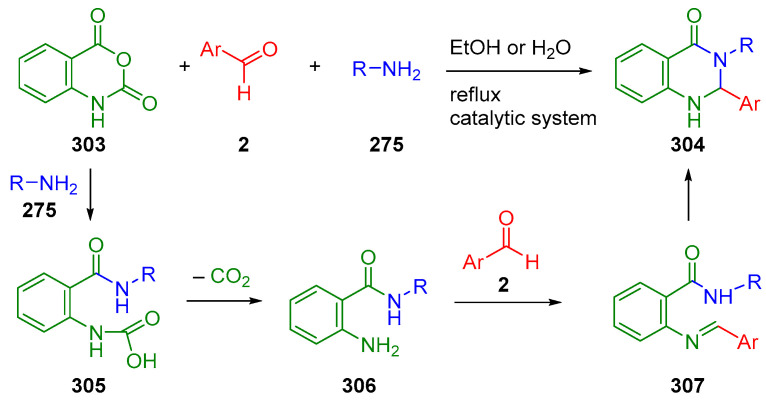
The reaction of isatoic anhydride with aldehydes and amines.

The reaction of isatoic anhydride (**303**), aldehydes (**2**), and amines (**275**) or ammonia yields 2,3-dihydroquinazolin-4(1*H*)-ones (**304**) in one-pot. However, it cannot be considered a real CCR, as isatoic anhydride (**303**) has no enolisable positions [156]. The reaction takes place by a different mechanism than the CCR: an attack of the amine (**275**) on the anhydride (**303**) with the loss of a molecule of CO_2_ results in an intermediate ortho-amino amide (**306**). This reacts with the aldehyde (**2**) to form an imine (**307**) that readily cyclises to yield the final pyrimidine ring (**304**; Scheme 92). The reaction is facilitated by different catalytic systems, such as silica/sulfuric acid [156], alum [157], sulfuric acid functionalised dendritic fibrous nano-silica 1 (KCC-1) [158], ionic liquid [159], triethanolamine and NaCl in water [160], and a silica-supported iron complex [161].

**Scheme 93:**
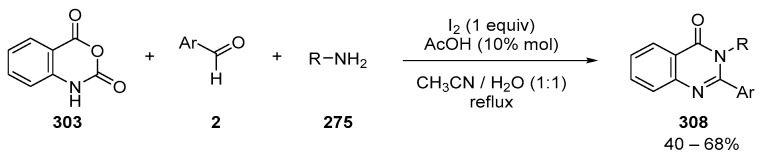
Dabiri’s three-component synthesis of quinazolinones [163].

Quinazolin-4(3*H*)-ones (**308**) can also be directly obtained in a sequence protocol in which ceric ammonium nitrate [162] or I_2_ are used as oxidants (Scheme 93) [163,164].

An efficient synthesis of quinazolin-4(3*H*)-ones (**308**) in PEG-400, under air and using formamide as an ammonia precursor, was recently published by Pal and co-workers [165]. This methodology was successfully used for the synthesis of alkaloids leutonin B and E, bouchardatine, and 8-norrutaecarpine.

A number of variants of these reactions of isatoic anhydride have been published and this chemistry has been recently reviewed [166].

## 7. Miscellaneous

**Scheme 94:**
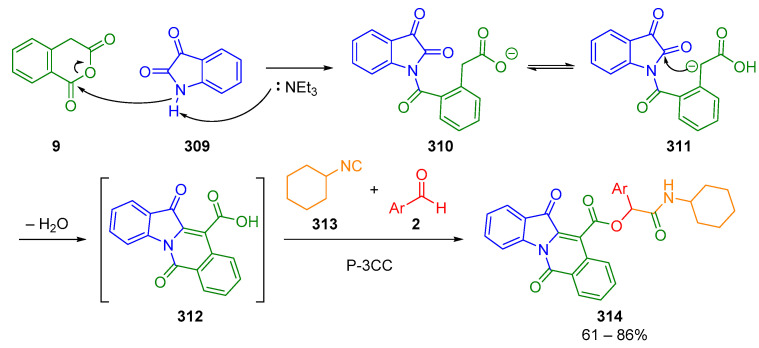
CCR/P-3CC for the synthesis of indolo[1,2-*b*]isoquinolines.

An interesting four-component combination of a Castagnoli–Cushman-type reaction and a Passerini condensation (P-3CC) was recently published by Rahmati (Scheme 94) [167]. In this reaction, isatin (**309**), HPA (**9**), cyclohexyl isocyanide (**313**), and an aldehyde (**2**) were combined to produce indolo[1,2-*b*]isoquinoline derivatives (**314**) in one pot.

**Scheme 95:**
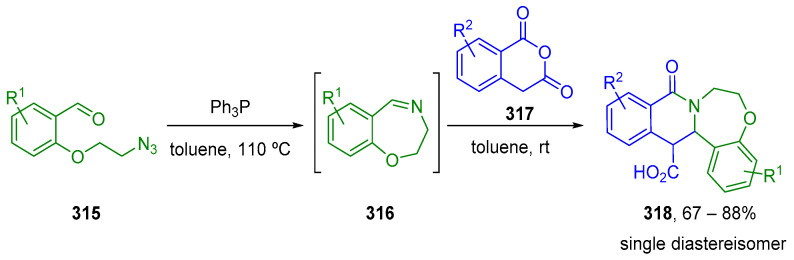
Staudinger/aza-Wittig/Castagnoli–Cushman reaction.

In addition, a one-pot Staudinger/aza-Wittig/Castagnoli–Cushman sequence for a series of azido aldehydes (**315**) and homophthalic anhydrides (**317**) readily affords natural product-like heterocycles (**318**; Scheme 95) [168].

## 8. Conclusions and Future Research Directions

The reaction of cyclic anhydrides and imines leading to lactams has been known for more than 50 years. In this time period, our understanding of the reaction mechanism has improved, and new versions of the original reaction have been developed. The limits of the synthetic potential of this reaction are still unknown, and new applications are published every year. Particularly interesting are the three- and four-component CCRs, which allow for the combinatorial synthesis of complex molecules from simple starting materials. In addition, stereochemical control and the use of chiral catalysts allow access to new biologically relevant molecules, which makes CCR a very useful tool in medicinal chemistry.
